# Cerebral *Plasmodium falciparum* malaria: The role of PfEMP1 in its pathogenesis and immunity, and PfEMP1‐based vaccines to prevent it

**DOI:** 10.1111/imr.12807

**Published:** 2019-09-27

**Authors:** Anja Ramstedt Jensen, Yvonne Adams, Lars Hviid

**Affiliations:** ^1^ Centre for Medical Parasitology at Department of Immunology and Microbiology Faculty of Health and Medical Sciences University of Copenhagen Copenhagen Denmark; ^2^ Department of Infectious Diseases Rigshospitalet Copenhagen Denmark

**Keywords:** antibodies, cerebral malaria, immunity, PfEMP1, *Plasmodium falciparum*, vaccine

## Abstract

Malaria, a mosquito‐borne infectious disease caused by parasites of the genus *Plasmodium* continues to be a major health problem worldwide. The unicellular *Plasmodium*‐parasites have the unique capacity to infect and replicate within host erythrocytes. By expressing variant surface antigens *Plasmodium falciparum* has evolved to avoid protective immune responses; as a result in endemic areas anti‐malaria immunity develops gradually over many years of multiple and repeated infections. We are studying the role of *Plasmodium falciparum* erythrocyte membrane protein 1 (PfEMP1) expressed by asexual stages of *P*.* falciparum* responsible for the pathogenicity of severe malaria. The immunopathology of *falciparum* malaria has been linked to cyto‐adhesion of infected erythrocytes to specific host receptors. A greater appreciation of the PfEMP1 molecules important for the development of protective immunity and immunopathology is a prerequisite for the rational discovery and development of a safe and protective anti‐disease malaria vaccine. Here we review the role of ICAM‐1 and EPCR receptor adhering *falciparum‐*parasites in the development of severe malaria; we discuss our current research to understand the factors involved in the pathogenesis of cerebral malaria and the feasibility of developing a vaccine targeted specifically to prevent this disease.

## 1 INTRODUCTION

Infection with *Plasmodium falciparum* parasites causes the most severe form of malaria that is responsible for essentially all malaria‐related deaths. The ability of *P*.* falciparum*‐infected erythrocytes (IEs) to adhere efficiently to host vascular receptors sets this parasite aside from the other malaria parasites infecting humans, and is generally considered an important reason why *P*.* falciparum* malaria is particularly dangerous.

IE adhesion is called sequestration when the IEs stick to tissue‐bound receptors, rosetting when they stick to uninfected erythrocytes, and clumping when the IEs stick to each other. It can lead to circulatory disturbances, vascular occlusion, and inflammation. In all cases, the IEs interact with host receptors via members of parasite‐encoded antigens displayed on the IE surface. These antigens belong mainly—if not exclusively—to products of several multigene families. Prominent among them—and by far the best studied—is *Plasmodium falciparum* erythrocyte membrane protein 1 (PfEMP1), encoded by the clonally variant *var* gene family with approximately 60 members per parasite genome. This review focuses on PfEMP1, the putative role of this antigen family in the development of one of the most severe forms of malaria called cerebral malaria (CM) and in acquired immunity to CM, and finally on the prospect of a PfEMP1‐based vaccine to prevent this often fatal complication. Before discussing each of these aspects it is necessary to recapitulate briefly the parasite life cycle, as it is important for appreciating the sections that follow.

### 1.1 The parasite multiplication cycle

**Figure 1:**
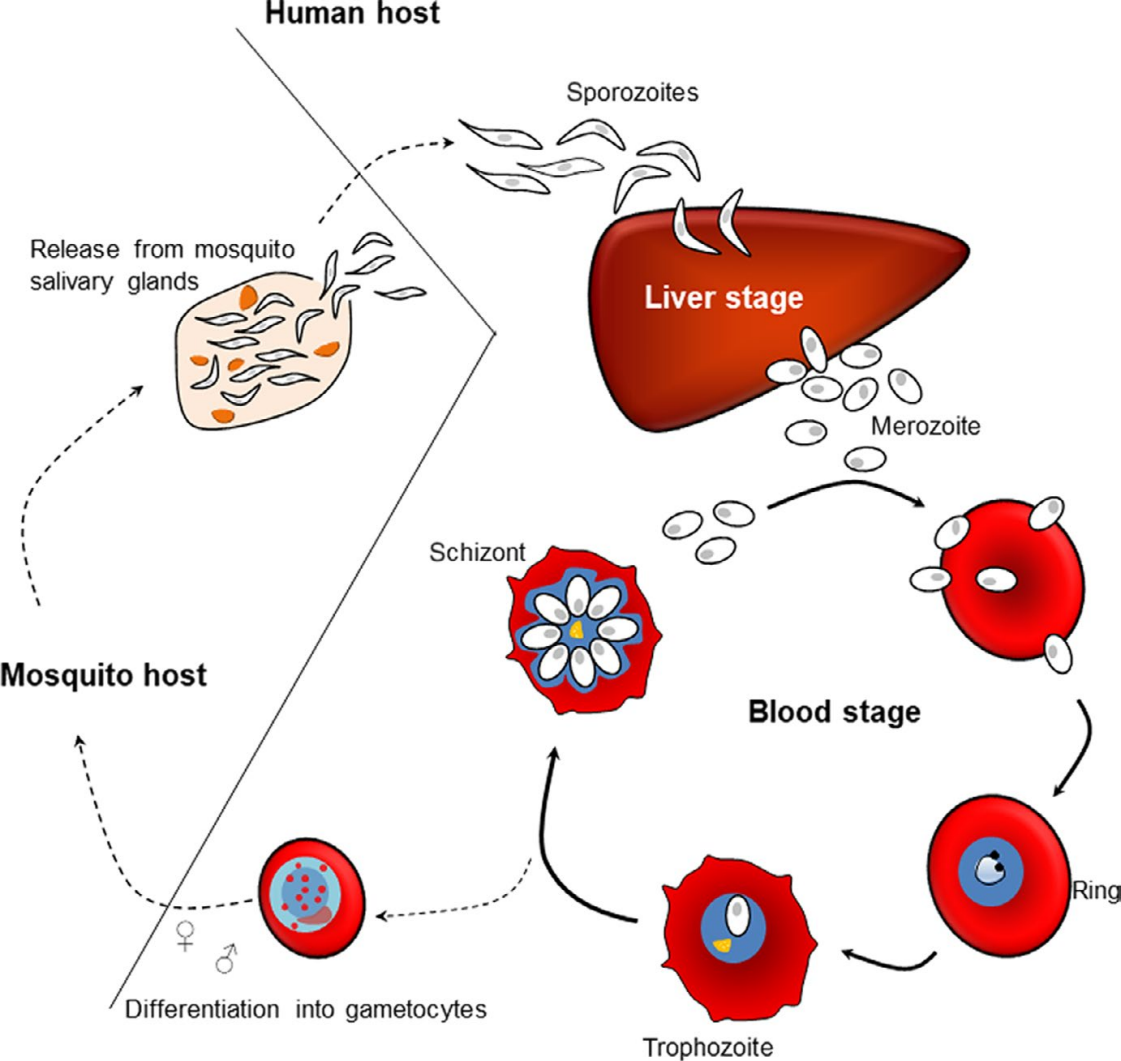
Life cycle of *Plasmodium falciparum*. Human infection with *P*.* falciparum* parasites is initiated when an infected female *Anopheles* spp. mosquito injects sporozoites during a blood meal. Sporozoites transit from the host peripheral circulation to the liver, where they infect hepatocytes. The liver stage is asymptomatic and lasts for approximately 1 wk. Eventually, the infected liver cells rupture to release extracellular merozoites into the host circulation. The merozoites invade erythrocytes, thereby initiating the asexual blood stage of the infection, which causes all the clinical symptoms of malaria. Once inside the erythrocyte, the merozoite undergoes a series of divisions (schizogony) over a period of 48 h, following which daughter merozoites are released to infect new erythrocytes. Some asexual parasites do not undergo schizogony, but develop into sexual precursors (gametocytes), which can be taken up by mosquitoes during a blood meal to complete the life cycle

*Plasmodium falciparum* has a complex life cycle that involves two hosts (humans and *Anopheles* spp. mosquitoes), and several developmental stages in each host (Figure 1). The human part of the multiplication cycle, which is asexual, is initiated when a *P*.* falciparum*‐infected female mosquito injects sporozoite‐stage parasites into the skin while it is feeding for blood. The extracellular sporozoites rapidly transit via the peripheral circulation from the skin to the liver, where they infect hepatocytes. The liver stage is asymptomatic and lasts for approximately 1 week, during which time the intrahepatic parasite multiplies, resulting in a (pre‐ or extraerythrocytic) schizont that consists of at least 30 000 daughter parasites. These, now called (pre‐ or extraerythrocytic) merozoites leave the infected hepatocyte and enter the blood circulation. The merozoites rapidly infect erythrocytes, an event that marks the beginning of the intraerythrocytic multiplication cycle. This part of the life cycle continues until the infection is controlled by either immunity or chemotherapy, or until the host dies. Each round of the intraerythrocytic cycle lasts approximately 48 hours. During each, the newly invaded merozoite rapidly transforms to a trophozoite (the early trophozoite is often called a ring‐stage parasite, because of the prominent vacuole) that undergoes three to five mitotic divisions, resulting in a schizont. At the end of the intraerythrocytic cycle, the IE ruptures and the released (erythrocytic) merozoites rapidly invade new erythrocytes. Some of the newly invaded merozoites develop into male or female gametocytes rather than continuing the asexual multiplication cycle. The gametocytes do not divide, but remain inside the erythrocyte until taken up by a blood‐feeding mosquito, where sexual reproduction and further asexual multiplication steps complete the parasite life cycle.^1^, ^2^, ^3^

## 2 THE *P*.* falciparum* ERYTHROCYTE MEMBRANE PROTEIN 1 ANTIGENS

The expression of PfEMP1 is largely (but not exlusively^4^) restricted to the intraerythrocytic blood stages of the infection, where these high‐molecular weight proteins mediate IE adhesion to a variety of host receptors. Intracellular PfEMP1 can be detected a few hours after the merozoite has invaded an erythrocyte, whereas IE surface expression starts about 16 hours postinvasion.^5^ The PfEMP1 surface expression reaches a plateau about 8 hours later, and starts to decrease during the final hours of the 48‐hour cycle,^6^ since export of PfEMP1 to the IE surface stops 30‐36 hours postinvasion.^7^

### 2.1 PfEMP1 structure

The members of the PfEMP1 family are high‐molecular weight proteins (200‐450 kD), encoded by approximately 60 two‐exon *var* genes per haploid *P*.* falciparum* genome.^5^ The extracellular part of PfEMP1, which is encoded by exon I, is composed of cysteine‐rich interdomain regions (CIDRs) and 2‐10 Duffy‐binding‐like (DBL) domains. These DBL and CIDR domains can be divided into seven (α, β, γ, δ, ε, ξ, x) and three (α, β, γ) main sequence classes, respectively, each with many further subdivisions.^8^ While exon I is characterized by extensive intraclonal (within single genomes) and interclonal (between genomes) sequence variation, the short transmembrane domain and the acidic intracellular terminal segment (ATS) are encoded by the relatively conserved exon II.^8^

**Figure 2:**
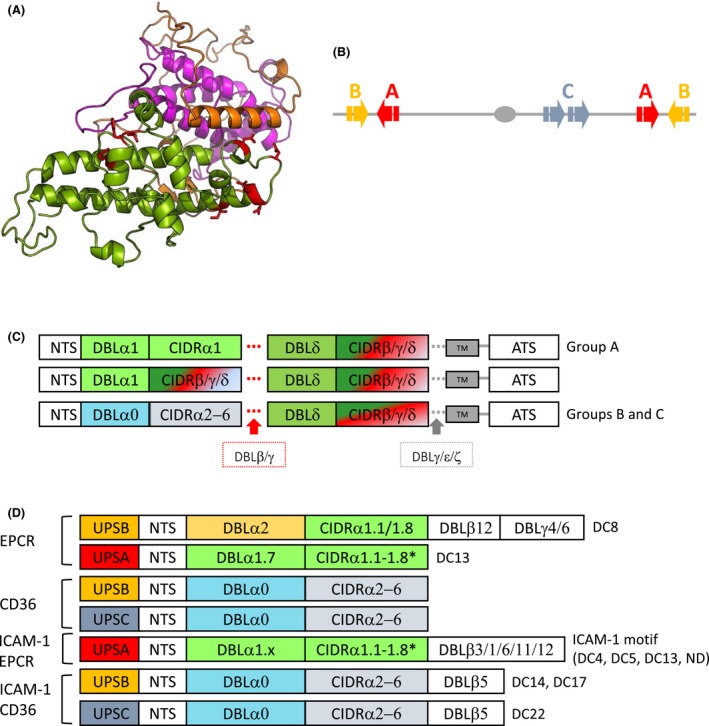
*var* genes and PfEMP1 structure. (A), Modeled structure of PFD1235w DBLβ. The structure consists of subdomain 1 (S1, orange) with mixed helix‐sheet structure and two helix bundles; subdomain 2 (S2 magenta) and subdomain 3 (S3 green).^12^ The ICAM‐1 binding site of PFD1235w in S3 is indicated in red.^14^, ^18^ (B), Group A and B *var* genes are located in subtelomeric regions of all chromosomes but are transcribed in opposite directions, whereas Group C *var* genes are found in central chromosomal regions. (C), PfEMP1 proteins are composed of different subtypes of DBL and CIDR domains. Groups B and C PfEMP1 predominantly have a four‐domain structure, while larger PfEMP1 proteins have additional DBL domains following the first or second DBL‐CIDR domains. (D), *Plasmodium falciparum* genomes encode tandem domain cassettes (DC) that are linked to different known adhesion phenotypes as indicated. DC8 is a chimeric gene between a group A and a group B *var* gene. Redrawn and modified from Hviid and Jensen^17^

The PfEMP1 CIDR domains are characterized by conserved cysteine‐rich motifs,^9^ while the DBL domains are homologous to *P*.* falciparum* EBA‐175 adhesive domains and to the Duffy‐binding proteins of *P*.* vivax* and *P*.* knowlesi*.^10^ The DBL domains are composed of three structural subdomains (Figure 2A), which have a mixed helix‐sheet structure (S1) or consist of helix bundles (S2 and S3).^11^ The subdomains are held together by disulphide bonds between conserved cysteine residues,^12^ whereas the α‐helices of the CIDR and DBL domains are connected by flexible and/or ordered loops. The functional specificity of different PfEMP1 proteins often (but not always) depends on these highly variable loops.^12^, ^13^, ^14^

Despite the sequence variability, PfEMP1 proteins can be grouped according to their chromosomal location, upstream promotor sequence (*ups*), and direction of transcription of the *var* genes encoding them.^15^ Group A (10 genes in *P*.* falciparum* 3D7), Group B (22 genes), and Group B/A *var* genes (4 genes) are all found in the subtelomeric regions of chromosomes. Group A genes are transcribed toward the telomere, whereas Group B and B/A *var* genes are transcribed toward the centromere (Figure 2B). Group C (13 genes in *P*.* falciparum* 3D7) and Group B/C *var* genes (9 genes) are typically found in internal regions of chromosome 4, 7, 8 and 12.^5^, ^8^, ^15^, ^16^ All PfEMP1 subfamilies except two (Type 3 and VAR2CSA) have a head structure at their N‐terminus that is composed of semiconserved DBLα domain and a CIDR domain.^5^ This is followed by a second and more diverse DBL‐CIDR pair in most PfEMP1 proteins belonging to Group B, B/C, and C. Group A and B/A PfEMP1 proteins are composed of a total of 7‐10 extracellular domains, including additional DBL domains upstream and/or downstream of the second DBL‐CIDR pair (Figure 2C).

The combination of DBL and CIDR subtypes in different PfEMP1 proteins is non‐random, and has led to the identification of 21 domain trains called domain cassettes (DCs).^8^ The DCs are defined as *var* gene sequences encoding two or more DBL or CIDR domains with subclasses that can be predicted from each other, and they often predict the receptor specificity of the encoded PfEMP1 (Figure 2D).^17^ DC4 (DBLα1.1/1.4‐CIDRα.6‐DBLβ3),^18^ DC8 (DBLα2‐CIDRα1.1‐DBLβ12‐DBLγ4/6), and DC13 (DBLα1.7 and CIDRα1.4)^8^ are the DCs studied most extensively.

Most PfEMP1 appear to be elongated and are rigid molecules with a zigzag shape and a length of about 30 nm,^19^, ^20^ although VAR2CSA‐type PfEMP1 assume a more compact and globular shape with a diameter of approximately 20 nm.^21^, ^22^

### 2.2 PfEMP1 function

The primary function of the PfEMP1 proteins is to mediate adhesion of IEs to host receptors in the vasculature.^17^, ^23^ This sequestration is vital to the parasites, as it allows mature IEs (misshapen and rigid because of the parasites growing inside them) to avoid the spleen passage, where they would be filtered and destroyed.^24^, ^25^ A range of vascular surface proteins and carbohydrates can serve as IE adhesion receptors, including CD36,^26^ intercellular adhesion molecule 1 (ICAM‐1),^27^ endothelial protein C receptor (EPCR),^28^ oncofetal chondroitin sulphate (CSA),^29^, ^30^ and ABO blood group antigens.^31^ The expression of IE adhesion receptors varies between different vascular beds, and is often regulated by cytokines.^32^, ^33^

Many different PfEMP1 proteins appear to have specificity for the same receptor, and this to some extent corresponds to the structurally defined PfEMP1 groups and domain subclasses mentioned above.^8^, ^15^, ^16^ Thus, subclasses of DBLβ domains found in Group A, B, and C PfEMP1 bind ICAM‐1.^14^, ^18^, ^34^, ^35^ CIDRα1 domains from Group A and B/A PfEMP1 bind EPCR,^13^, ^28^ whereas Group B and C contain CIDRα2‐6 domains that bind CD36 (Figure 2D).^36^, ^37^ Finally, Group E (VAR2CSA‐type) PfEMP1, which appear responsible for IE accumulation in the placenta, have unique affinity for CSA.^30^, ^38^, ^39^ It is not very surprising that several of these large, multidomain proteins contain domains that can simultaneously mediate adhesion to several host receptors.^14^, ^35^, ^40^, ^41^ Examples include PfEMP1 variants concurrently interacting with ICAM‐1 and EPCR,^14^, ^41^ with ICAM‐1 and CD36,^35^ or variants capable of distinct receptor‐ligand interactions with endothelial cells and uninfected erythrocytes.^42^

### 2.3 Clonal antigenic variation controlling PfEMP1 expression

Aside from an exception of unresolved biological significance,^43^ only one PfEMP1 variant is expressed on the surface of a given IE at any given time,^44^ but the expressed variant may change from one 48‐h cycle to the next. The ability to switch from the expression of one PfEMP1 variant to another (called clonal antigenic variation) acts as a key to the pathogenicity of *P*.* falciparum* parasites and is a major determinant of the characteristic chronicity of untreated infections.^45^ The genetic processes governing clonal antigenic variation in *P*.* falciparum* parasites are complex, and will not be described here, as they have been recently reviewed in detail elsewhere.^46^ However, the switching pattern does not appear to be fixed, but rather seems to follow a loose hierarchy determined by variations in the intrinsic switching (on/off) rates of individual *var* genes.^47^, ^48^, ^49^, ^50^

### 2.4 PfEMP1 expression on the infected erythrocyte surface

**Figure 3:**
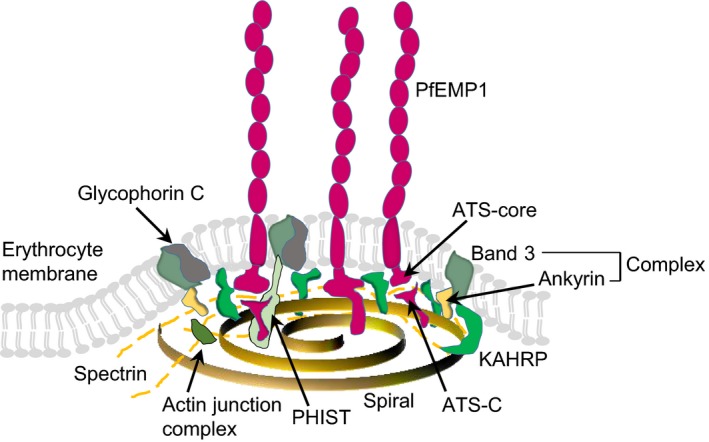
PfEMP1 presentation on knobs on the IE surface. KAHRP form the base of the knob complex. An average of three PfEMP1 molecules are located at the tip of each knob.^58^ KAHRP binding to spectrin is necessary for the formation of the spiral structure, but KAHRP itself does not appear to be a component of the spiral.^54^ PHIST protein may connect to PfEMP1 and the cytoskeleton.^56^ Redrawn and modified from Cutts et al^53^

The expression of PfEMP1 on the IE surface is confined to membrane protrusions called knobs.^51^ Formation of knobs involves multiple host and parasite molecules in addition to PfEMP1, such as the parasite‐encoded knob‐associated, histidine‐rich protein (KAHRP) which multimerizes into a five‐molecule spiral cone‐like structure linked to erythrocyte cytoskeleton spectrin‐ankyrin complexes (Figure 3).^52^, ^53^, ^54^

The nascent PfEMP1 molecules are exported to the IE surface via a complex, multistep process that has recently been reviewed in detail elsewhere.^55^ Their ATS domains bind to the cytoskeleton via LyMP (a member of the PHIST [*Plasmodium* helical interspersed subtelomeric] protein family (Figure 3)).^53^, ^56^, ^57^ Recent data suggest that the exported PfEMP1 molecules are delivered to the IE membrane away from the knobs, and then moved laterally and assembled into the knobs.^54^

The knobs appear on the IE surface approximately 16 hours postinvasion, peak in density about 20 hours later, followed by a slight decrease in density toward the end of the intraerythrocytic 48‐h cycle.^6^ Each knob appears to accommodate less than a handful of PfEMP1 molecules, which are expressed in a cluster near the tip of the knob.^58^ The reason for the clustered and knob‐confined display of PfEMP1 on the IE surface is not fully understood. However, it likely includes optimization for adhesion, as the surface knob density appears to vary with the PfEMP1 expressed and may be modulated by immunity, and because knob‐less IEs generally have reduced PfEMP1 expression and do not adhere well.^6^, ^59^, ^60^, ^61^ Consistent with that, disruption of the gene encoding KAHRP leads to disappearance of knobs, decreased PfEMP1 expression, and reduced IE adhesiveness.^62^, ^63^, ^64^ Disruption of the gene encoding LyMP causes a similar decrease in IE adhesiveness, although expression of both PfEMP1 and knobs remain at wildtype levels.^57^

Altogether, these findings suggest that the overall IE adhesiveness is the net result of which PfEMP1 is expressed (quality), how much of it is expressed (quantity), and how it is expressed (topology). This conclusion is supported by studies linking the protective effects of haemoglobinopathies such as HbC, HbS, and α‐thalassemia to an impaired ability of *P*.* falciparum* parasites to adequately remodel the erythrocyte cytoskeleton and display PfEMP1 in these aberrant erythrocytes.^65^, ^66^, ^67^, ^68^

## 3 CEREBRAL MALARIA

Cerebral *P*.* falciparum* malaria (CM) is defined by the World Health Organization (WHO) as deep and unarousable coma that persists for more than 1 hour after a seizure, irrespective of anticonvulsant medication, in a patient with peripheral *P*.* falciparum* parasitemia and without another cause of encephalopathy.^69^ It is estimated that around 1% of children infected with *P*.* falciparum* develop CM, which has a very high mortality despite treatment, with up to 75% of deaths occurring within the first 24 hours postadmission.^70^, ^71^, ^72^ CM is a leading cause of the estimated >400 000 deaths due to malaria each year^73^ despite the fact that this clinical definition of CM may lead to misclassification in as many as one in four cases.^74^, ^75^

In sub‐Saharan Africa, CM mostly affects children under 5 years of age who have only partial acquired immunity to *P*.* falciparum* infection. Several studies have shown seasonal and transmission intensity‐dependent differences in the frequency of CM,^76^, ^77^ suggesting that the level of acquired immunity is an important determinant of CM susceptibility.^78^ This is supported by the finding that CM is mainly seen among older children and adults in Southeast Asia, where malaria transmission is less intense than in sub‐Saharan Africa.^79^

There are significant differences in the pattern of vital organ dysfunction between African children and Southeast Asian adults with CM.^70^, ^80^, ^81^, ^82^ In children, CM coincides with a period of rapid brain growth and physiologic changes of the blood‐brain‐barrier (BBB) that may account for some of these differences.^83^ Although CM in children generally has lower mortality than in adults under otherwise comparable conditions, seizures are more frequent and rapid, and CM in children is more often associated with anemia and neurocognitive sequelae.^83^, ^84^ Retinal vessel changes, ring hemorrhages, and accumulation of inflammatory cells in the brain microvasculature are also more frequent, and hypoglycemia is one of the most common concomitant complication in pediatric CM cases.^83^, ^84^, ^85^

In the following sections, we will focus on CM as it occurs among children in Africa because this is the population most affected, and also because most of the available knowledge about CM stems from studies of African children (Box 1).

Box 1 **Animal models of *Plasmodium falciparum* malaria**

A detailed analysis of the intracerebral pathogenesis and pathology in CM comes primarily from postmortem studies. For this reason, infections of various strains of mice with *P*.* berghei* ANKA (PbA) is a popular in vivo model to dissect the mechanisms of CM. PbA infection can induce a condition known as experimental cerebral malaria (ECM), which recapitulates some—but certainly not all—features of CM. This has led to considerable scepticism regarding the utility of this murine model.^364^, ^365^

In both humans and mice, the clinical signs of CM progress from seizures, ataxia, and paralysis to coma and eventually death.^366^ However, although cerebral IE sequestration is a prominent feature of CM,^364^ it is a minor feature of ECM.^367^, ^368^ Intravascular accumulation of platelets and immune cells has been observed in ECM, and CD8^+^ cells appear central to ECM pathogenesis. 369, 370, 371, 372 The picture is less clear in CM, where some studies have reported infiltration of leukocytes and platelets within the brain microvasculature,^96^, ^173^ whereas others did not find that.^195^, ^373^ Intravascular accumulation of monocytes has also been reported, but there is little evidence of high numbers of CD8^+^ T‐cell accumulation in human CM.

ECM is characterized by a prominent pro‐inflammatory cytokine response with high levels of IFN‐γ and TNF‐α, which results in upregulation of activation markers including ICAM‐1, VCAM‐1, and E‐selectin.^374^ Although TNF‐α ‐deficient mice are also susceptible to PbA‐induced ECM,^375^ ICAM‐1 must be present for ECM to develop.^376^ Although inflammatory changes in the brain are lower in CM, elevated concentrations of circulating pro‐inflammatory cytokines are characteristic.^377^, ^378^ This contributes to a marked increase in the expression of endothelial cell adhesion molecules,^106^, ^144^ and IEs and ICAM‐1 co‐localize in cerebral vessels postmortem.^106^ Of particular relevance to the present text, none of the genomes of rodent malaria parasite encodes PfEMP1‐like molecules, and we therefore direct interested readers to comprehensive ECM reviews elsewhere.^379^, ^380^

### 3.1 Subtypes of CM

Retinopathy, characterized by retinal hemorrhages, papilledema, retinal whitening, and vessel color changes, is the most specific clinical diagnostic sign of CM,^86^, ^87^, ^88^ because it directly reflects the cerebral sequestration of IEs and the pathological processes occurring in the brain.^88^, ^89^, ^90^ It thus allows distinction between children with coma caused by cerebral IE sequestration and those, whose coma has other causes but fulfil the abovementioned WHO criteria for CM. The number of retinal hemorrhages prior to death correlates with the density of hemorrhages in the brain at mortem.^91^ Retinopathy has therefore been suggested to reflect the spectrum of CM severity^86^, ^92^, ^93^ as patients with retinopathy have higher mortality than those without.^94^, ^95^

Pediatric CM can also be divided into two types based on clinical and pathological findings. CM1 (25% of cases) is characterized by IE sequestration in the cerebral microvasculature, rapid disease progression, limited organ pathology, and high splenic parasitemia. CM2 is characterized by more prolonged illness and parasite proliferation, with evidence of vascular brain pathology alongside endothelial dysfunction, activation of coagulation, ring hemorrhages, fibrin‐platelet thrombi, and accumulation of monocytes in addition to sequestered IEs.^75^, ^82^, ^96^ The proportion of retinal capillaries with extraerythrocytic hemozoin predicts CM1 and CM2.^75^, ^97^

Increased intracranial pressure with brain swelling and perivascular edema are strongly associated with CM severity.^88^ This is because the increased volume of the swollen brain can cause brainstem herniation leading to death by respiratory arrest.^98^, ^99^ These manifestations are partly due to breakdown of the blood‐brain barrier.

### 3.2 The blood‐brain barrier

The BBB is vital for normal brain function and constitutes a physiological barrier that separates the brain and the cerebrospinal fluid from the rest of the body.^100^ The BBB acts as a semipermeable cellular interface that tightly regulates the bidirectional transcellular molecular transport (of glucose, amino acids, transferrin, charged plasma proteins etc) between the blood and the brain parenchyma that is required to maintain cerebral homeostasis.

The BBB components include microvascular endothelial cells forming a continuous barrier through tight junctions, a basement membrane, pericytes, and astrocytes that are in direct contact with neurons and microglia. This composition is critical to minimize local inflammation and neuronal damage.^101^ Brain endothelial cells are structurally and functionally different from endothelial cells in other organs. In particular, they have intercellular tight and adherens junctions, which normally impede passive paracellular diffusion of small and large molecules and prevent infiltration of blood cells into the brain parenchyma.

Disruption of the BBB is common in diseases of the central nervous system.^102^ It is also a feature of CM,^103^ where it is thought to be the result of endothelial inflammation in the brain, caused by accumulation and sequestration of IEs, leukocytes, and platelets.^85^, ^104^, ^105^ Focal loss of the endothelial intercellular junctions that are central to the maintenance of BBB integrity has been observed in vessels containing sequestered IEs.^103^, ^104^, ^106^, ^107^, ^108^ The finding of decreased transendothelial resistance and changes in the expression of proteins that make up these junctions in brain endothelial cells exposed to IEs in vitro is consistent with these observations.^109^, ^110^ Numerous parasite and host factors that have been implicated in CM pathology can affect the permeability of the BBB. These include hemozoin‐induced matrix metalloproteases (MMP), angiopoietins, sphingosine‐1‐phosphate, nitric oxide, platelet‐activating factor, several cytokines (IL‐1α, IL‐1β, IL‐6, TNFα), and a number of other factors.^91^, ^111^, ^112^, ^113^, ^114^, ^115^, ^116^, ^117^ As an example, MMP targets structural proteins of the basal lamina (fibronectin, laminin, heparan sulfate) and tight junction proteins (ZO‐1, ZO‐2, claudin‐5), which is known to cause breakdown of tight junctions, increased paracellular leak, and opening of the BBB during ishemic and inflammatory insults. Another protein, histidine‐rich protein‐2 (HRP‐2) that is released at the time of schizont rupture,^118^ can activate the innate immune system via NLRP3 inflammasome activation. The ensuing compromising of tight junction integrity and IL‐1β‐ and MyD88‐dependent increased vascular permeability has been proposed to promote CM pathogenesis.^119^ In support of this, HRP‐2 has been shown to line the endothelial walls of blood vessels,^120^ particularly in retinopathy‐positive CM patients.^121^ Once the BBB is disrupted, leukocytes, cytokines, chemokines, and soluble parasite products may enter the brain parenchyma to activate the microglia and damage astrocytes and neurons, causing neuro‐inflammation and coma.^122^

### 3.3 Endothelial activation

Endothelial inflammation is a characteristic feature of *P*.* falciparum* malaria and correlates with disease severity in general and CM in particular.^123^, ^124^, ^125^, ^126^, ^127^ The inflammation may be induced directly by IEs adhering to the endothelium, or indirectly by inflammatory host and parasite products (such as IE membrane components, HRP‐2, etc).^128^, ^129^, ^130^, ^131^, ^132^ However, activation may also occur independent of IEs as there is also evidence of generalized endothelial inflammation at sites devoid of IE sequestration.^110^, ^133^, ^134^, ^135^

Endothelial cells derived from Malawian children with CM have been shown to express particularly high levels of parasite and platelet receptors, to produce many endothelial microvesicles, release high levels of pro‐inflammatory cytokines (including TNFα and IFNγ), and to be highly prone to undergo apoptosis.^134^, ^136^, ^137^ It seems likely that IEs may be involved, as they can induce apoptosis in primary brain endothelial cells, including cells from the brain,^138^ and cellular apoptosis has been suggested to cause increased endothelial permeability.^138^, ^139^, ^140^, ^141^

Activated brain endothelial cells are known to express high levels of a number of potential IE receptors (ie, ICAM‐1, VCAM‐1, P‐selectin, and E‐selectin), exocytose Weibel‐Palade bodies, release microvesicles, vascular endothelial growth factor (VEGF), and soluble cell adhesion molecules (ie, sICAM‐1), and to show breakdown of tight junctions.^142^, ^143^, ^144^, ^145^, ^146^ Three bioactive molecules are released from the Weibel‐Palade bodies, P‐selectin (recruiting leukocytes), von Willebrand Factor (vWF; binding platelets), and Angiopoetin (Ang)‐2. Ang‐1 and Ang‐2 are critical soluble regulators of endothelial activation and integrity, and levels of Ang‐1 and Ang‐2 have been described as reliable biomarkers of CM.^147^ Ang‐2 is a vessel‐destabilizing molecule that increases vascular permeability and facilitates endothelial activation by counteracting the action of Ang‐1 by displacing Ang‐1 from the receptor.^148^, ^149^, ^150^ Ang‐1 conversely mediates activation of the Tie‐2 receptors on endothelial cells. This inhibits apoptosis, reduces expression of ICAM‐1, VCAM‐1, and E‐selectin, promotes NO synthesis, and increases the expression of endothelial tight junctions.^151^, ^152^, ^153^, ^154^ It thus acts as a promoter of endothelial cell quiescence and survival. Release of Ang‐2 from Weibel‐Palade bodies increases the Ang‐2/Ang‐1 ratio and thus endothelial responsiveness. Increased concentration of Ang‐2 with decreased levels of Ang‐1 has been associated with development of severe malaria in several studies,^113^, ^114^, ^115^, ^147^, ^155^ and children with retinopathy‐positive CM have higher levels of Ang‐2, Ang‐2/Ang‐1, soluble Tie‐2, von Willebrand Factor, VEGF, and sICAM‐1, and lower levels of Ang‐1, compared to CM patients without retinopathy.^114^ Both Ang‐1 and Ang‐2 are regulated by nitric oxide (NO) produced in the endothelium from L‐arginine. NO causes vasorelaxation, downregulation of endothelial receptors, and reduces thrombosis.^156^ The bioavailability of NO is reduced in CM and this has been associated with fatal outcome.^157^, ^158^, ^159^ Low NO stimulates Weibel‐Palade body exocytosis and activation of endothelium, with increased Ang‐2 release from endothelial cells and expression of ICAM‐1 and VCAM‐1.^160^, ^161^ Impaired NO production thus disrupts the ang‐1/Tie‐2‐dependent signaling that maintains endothelial cell quiescence and vascular integrity.^159^, ^162^ This in turn promotes enhanced endothelial cell activation and cytoadhesion of IEs.^163^ All this notwithstanding, inhalation of NO was not found to reduce neurological defects or mortality in children with CM.^164^

von Willebrand Factor is synthesized by the endothelial cells, and some of the synthesized vWF is constitutively secreted into plasma, but most is stored within Weibel‐Palade bodies and only secreted following activation of the endothelial cell.^165^ vWF, particularly as large multimers, show enhanced binding to platelets and efficiently modulates aggregation of platelets.^166^ IEs can bind to platelets via P‐selectin, C1q receptors, and thrombospondin receptor (CD36), leading to formation of IE/platelet clumps.^167^, ^168^, ^169^ The significance of this is indicated by the observation that children who died of CM had more platelet build‐up in cerebral vessels than those dying of severe malarial anemia or non‐malarial encephalopathy.^170^ Platelet accumulation was particularly prominent at sites of IE sequestration. Platelet‐mediated IE clumping is thus likely to aggravate microvascular obstruction in CM, and release of tumor growth factor β from platelet granules may furthermore cause apoptosis of brain endothelial cells.^171^, ^172^ In addition, accumulation of platelets may enable transfer of CD36 to endothelial cells, thus potentially providing an additional IE receptor to brain endothelium, which normally expresses little or no CD36.^173^ Overall, the marked increase in plasma vWF levels in patients with severe malaria is likely to contribute to severe malaria pathogenesis.^114^, ^115^, ^166^, ^174^ Finally, activation of endothelium leads to increased shedding of microvesicles from the plasma membrane of cells.^175^ Endothelial microvesicles have been found in very high concentrations in children with CM, and their levels correlate with disease severity.^176^, ^177^ It has been proposed that these vesicles may contribute to excessive T‐cell activation and the immune pathogenesis of CM, as they express the molecules required for antigen presentation and T‐cell stimulation, such as β2‐microglobulin, MHC‐II, CD40, and ICOSL.^178^ In addition, increased concentrations of non‐endothelial microvesicles have been observed in CM, where they may also contribute to pathogenesis. Thus, the number of platelet‐derived microvesicles correlates with the depth of the coma and thrombocytopenia,^177^ and extracellular IE‐derived vesicles containing PfEMP1 (see below) can induce pro‐inflammatory cytokines in human primary monocytes.^179^, ^180^

## 4 PfEMP1 AND PATHOGENESIS OF CM

As mentioned above, *P*.* falciparum* parasites display PfEMP1 molecules on the surface of the erythrocytes they infect. From about 16 hours postinvasion, these high‐molecular weight variant parasite proteins efficiently mediate adhesion of the IEs to a range of host receptors,^181^, ^182^, ^183^, ^184^, ^185^ and this is the reason why only young, ring‐stage IEs are present in the peripheral circulation. It has long been speculated that PfEMP1‐mediated IE adhesion to specific receptors in key tissues and organs is an important determinant of clinical outcomes of *P*.* falciparum* infection. This hypothesis has been confirmed in the case of placental malaria, where the selective accumulation of IEs in the intervillous space is mediated by VAR2CSA‐type PfEMP1 with affinity for placental CSA.^30^, ^38^, ^186^ It is furthermore well established that protective immunity to placental malaria depends on acquisition of IgG to CSA‐adhering and VAR2CSA‐expressing IEs.^187^, ^188^, ^189^, ^190^ These findings have raised the hope that other specific PfEMP1 variants and host receptors may play similar and decisive roles in other forms of severe *P*.* falciparum* malaria, not least CM.^108^, ^191^, ^192^, ^193^

### 4.1 Sequestration of infected erythrocytes in the brain

IEs adhere to host endothelial receptors in the postcapillary venules of a number of organs, such as lungs, liver, intestine, brain, and the placenta.^194^, ^195^ This tissue‐specific sequestration causes circulatory disturbances and inflammation, and single‐ and multi‐organ pathology such as renal, liver, lung, and placental dysfunction, and CM.^196^, ^197^, ^198^, ^199^, ^200^, ^201^ Sequestration probably evolved to allow mature IEs, deformed by the parasites inside, to avoid destruction in the spleen.^24^ Identification of the parasite ligands, not least the specific PfEMP1 variants, which mediate IE sequestration in particular tissues—and the host receptors they bind to, has thus been a long‐standing and important research priority. CM research is no exception.

Many studies have reported links between severe malaria (including CM) in children and infection with parasites transcribing *var* genes encoding PfEMP1 proteins from Group A and B/A.^202^, ^203^, ^204^, ^205^, ^206^, ^207^, ^208^, ^209^, ^210^, ^211^ Other studies have narrowed the list of candidate genes to those having specific sequence signatures and/or encoding PfEMP1 variants with well‐defined receptor specificity.^28^, ^209^, ^210^, ^212^, ^213^, ^214^, ^215^, ^216^, ^217^ ICAM‐1 (CD54) was recognized as an endothelial IE receptor early on,^27^ and it has long been suspected to be important for the selective accumulation of IEs in the brain of CM patients.^106^, ^133^, ^144^, ^218^ In line with this, contact with IEs can lead to endothelial upregulation of ICAM‐1.^128^, ^129^, ^134^, ^219^ Furthermore, it has been reported that *P*.* falciparum* parasites isolated from African children with CM bind preferentially to ICAM‐1 in vitro.^220^ However, the opposite has also been reported,^221^ and isolates from Asian adult malaria patients do not appear to show preferential adhesion to ICAM‐1.^222^, ^223^ Finally, some studies have failed to find evidence of high ICAM‐1 expression in the brains of fatal CM victims.^220^, ^221^ Taken together, a complex picture regarding the relationship between ICAM‐1‐specific IE adhesion and CM pathogenesis emerges, although most of these data suggest that CM in (African) children is quite different from CM in (Asian) adults.^83^

IE adhesion to ICAM‐1 is mediated by PfEMP1 variants that can also bind to either EPCR or CD36.^14^, ^34^, ^35^, ^224^, ^225^ The former of these groups, exemplified by the *P*.* falciparum* 3D7 PfEMP1 PFD1235w,^18^ shows a clear association specifically with CM.^14^, ^226^, ^227^, ^228^, ^229^ PFD1235w belongs to Group A, and contains the domain cassette DC4 (Figure 2D). The DC4 family was originally identified by a search for orthologs of the *pfd1235w* gene in parasites from Ghanaian malaria patients, inspired by the link between PFD1235w and severe malaria.^202^, ^230^ The search resulted in a panel of genetically distinct parasites binding ICAM‐1 via the DBLβ3 domain of the DC4‐type PfEMP1 expressed on the IE surface.^18^ Sequence analysis of these domains identified a C‐terminal ICAM‐1‐binding motif (I[V/L]x_3_N[E]GG[P/A]xYx_27_GPPx_3_H).^14^ The motif, which is also present in some Group A PfEMP1 proteins outside DC4 (including some DC5‐ and DC13‐containing variants) and in a few Group B/A variants,^14^, ^18^ is restricted to DBLβ domains located immediately downstream of CIDR domains of the EPCR‐binding subtype.^13^, ^14^

Endothelial protein C receptor is the cognate receptor for PfEMP1 proteins containing domain cassette DC8 or DC13.^28^ DC13 is found among group A PfEMP1, whereas DC8 is found in Group B PfEMP1, and has evolved by recombination of ancestral Group A and B *var* genes.^209^ PfEMP1 variants containing DC8 or DC13 are common,^8^ and bind avidly to endothelial cells of lung, heart, and bone marrow.^231^ DC8‐containing PfEMP1 proteins tend to be among the first expressed in early childhood infections, indicating that they possess adhesion properties that confer a survival advantage to IEs in malaria‐naive children.^213^ In addition, *P*.* falciparum* parasites obtained from African children and Indian adults with severe malaria—including CM—transcribe DC8‐ and DC13‐encoding *var* genes at high levels.^209^, ^215^, ^216^, ^217^, ^226^, ^227^ Their relevance to CM pathogenesis is further indicated by studies showing that IEs selected for adhesion to brain endothelial cells preferentially express these domain cassettes.^213^, ^214^ Finally, expression of EPCR‐binding PfEMP1 variants from Group A have been linked to brain swelling,^228^ which is a major contributor to mortality in pediatric CM.^88^ The available evidence linking the EPCR‐adhering IE phenotype to severe malaria in general, and to CM in particular, is not completely unequivocal.^232^, ^233^, ^234^ As an example, a study of Kenyan children with CM did not find evidence supporting particular enrichment of DC8‐ or DC13‐containing PfEMP1 variants in children with retinopathy, a well‐established indicator of CM, despite finding the expected association between CM and transcription of *var* genes encoding Group A PfEMP1.^235^

As mentioned above, not all PfEMP1 variants capable of binding ICAM‐1 also bind EPCR. Indeed, all but one^236^ of the ICAM‐1‐binding DBLβ domains identified prior to the discovery of DC4 in Group A were found in Group B and Group C proteins.^34^, ^35^ Those PfEMP1 proteins appear to be under dual selection for adhesion to ICAM‐1 and CD36, as they all contain a CD36‐binding CIDRα domain upstream of the ICAM‐1‐binding DBLβ domain.^35^, ^36^, ^237^ This is not surprising, as CD36 is a very common IE adhesion receptor and most non‐placental *P*.* falciparum* isolates can bind to it.^30^, ^220^, ^221^ Affinity for CD36 is a feature of the majority of Group B and C PfEMP1 proteins,^35^, ^36^ but is not found in the Group A and B/A PfEMP1 that dominate in severe infections and in individuals with limited malaria immunity.^202^, ^204^, ^207^, ^209^, ^238^ Rather, CD36 binding is associated with uncomplicated malaria,^239^, ^240^ and appears to have evolved to mediate IE sequestration in tissues other than the brain, where CD36 is absent or only sparsely present.^135^, ^155^

Combining the above evidence, the adhesion phenotype that is most clearly related to CM is IEs expressing Group A PfEMP1 proteins (including DC4) that allow concomitant binding to both ICAM‐1 and EPCR (“double binders”).^14^ The association between severe disease (and in particular, CM) and IE affinity for either of these receptors alone is less clear. It is worth noting in that context that the EPCR‐binding CIDRα1 domain in DC8, and in some DC13, is not followed by an ICAM‐1‐binding DBL domain.^13^, ^14^ Finally, although several molecules other than those already mentioned have been implicated as IE adhesion receptors, including some that appear to be expressed on brain endothelium,^241^ none of them have been linked to disease severity.

### 4.2 Rosetting and clumping

Infected erythrocytes do not only adhere to the endothelium, but also to surrounding uninfected or infected erythrocytes. The former type of such aggregates, called rosettes, were first reported in the *P fragile*‐infected monkeys.^242^ The finding was quickly followed by studies demonstrating that the same phenotype was present in *P*.* falciparum*,^243^, ^244^ and it appears that most species of malarial parasites are capable of inducing rosettes,^245^ which is a complex phenotype involving multiple parasite and host molecules.^246^ Thus, several erythrocyte molecules, including complement receptor 1, heparan sulphate, and the ABO blood group antigens, appear to be involved as receptors.^247^, ^248^, ^249^, ^250^ On the parasite side, several ligands have been implicated, including members of several variant surface antigen families.^251^, ^252^

Infected erythrocytes isolated from patients with CM have been reported to form rosettes at significantly higher rates than IEs from patients with uncomplicated malaria.^253^, ^254^, ^255^, ^256^ This difference might contribute to occlusion of cerebral microvessels by rosettes, to cerebral sequestration of IEs that express PfEMP1 variants that can bind to host receptors shared by cerebral microvessels and erythrocytes,^257^, ^258^ and/or expressing PfEMP1 variants that allow binding to separate receptors on endothelium and on erythrocytes, respectively.^42^ With respect to the first of these possibilities, microvascular flow rates appear to be an important determinant for the clinical consequences of rosetting,^259^ similar to what appears to be the case for IE sequestration (see above). With respect to the last possibility, several PfEMP1 variants with capacity to bind to more than one receptor at the same time (mostly via different domains) have been described and linked to disease severity.^14^, ^40^, ^260^ However, while some studies have shown significant correlations between rosetting and severe malaria/CM,^253^, ^254^, ^261^, ^262^, ^263^ others have failed to find such a relationship.^264^, ^265^, ^266^ Although the reason for the discrepancy is not fully resolved, it may reflect genuine geographical differences between Africa (where the association is generally found) and Southeast Asia (where it usually is not). Similar geographic differences have been described for other receptor‐specific adhesive IE phenotypes and malaria severity.^220^, ^222^, ^267^, ^268^ If indeed such geographical variation exists, it may involve differences in the parasites, the hosts, and/or the transmission intensity. The relative contributions of these variables remain completely unresolved, however.

The receptor specificity of the PfEMP1 variant expressed is an obvious determinant of whether PfEMP1‐dependent rosetting occurs or not. However, soluble plasma factors that can bind to PfEMP1 may also be of pathologic significance. Thus, IE rosetting in vitro generally (but not always) requires the presence of plasma or serum in the assay, and several components have been implicated.^246^ The most studied of these is IgM, which can clearly enhance rosette formation.^20^, ^269^, ^270^ This function of IgM is independent of the antigen specificity of the antibody as it is mediated by the Fc rather than the Fab domains.^20^, ^271^ It furthermore requires a pentameric IgM conformation,^271^, ^272^, ^273^ probably because IgM augments rosette formation by cross‐linking multiple PfEMP1 molecules, thereby enhancing their combined avidity for the erythrocyte receptor involved.^20^, ^274^ Several of the approximately 60 PfEMP1 variants in a given genome contain domains that are able to bind IgM this way,^275^, ^276^ but the potential of this finding for identifying PfEMP1 proteins involved in the pathogenesis of severe malaria in general, and of CM in particular, is largely unknown. A single study recently pointed to the protease‐inhibitor α_2_‐macroglobulin as another important cofactor of rosetting,^277^ but the generality and clinical significance of this finding remains unclear.

IEs can also bind to other IEs via platelets (thrombocytes); a phenotype referred to as clumping.^167^ Clumping has been associated with severe malaria including CM in some, but not all studies.^169^, ^278^, ^279^ The platelet receptor involved appears to be gC1qR/HABP1/p32, and as this receptor is also present on cerebral microvascular endothelium, it provides a plausible link between clumping and C.^168^ However, to our knowledge it is not known whether IE affinity for gC1qR/HABP1/p32 is mediated by PfEMP1 or whether this adhesion phenotype is significantly involved in the pathogenesis of CM.

### 4.3 The role of blood flow on IE adhesion

Erythrocytes normally flow down the central line of a blood vessel,^280^, ^281^ but the deformation and enhanced stiffness of IEs cause them to marginate, thus bringing them into contact with adhesion receptors on the endothelial surface.^282^, ^283^ The distribution of cells, including IEs, in the blood stream is furthermore dependent on variables such as vessel diameter and plasma viscosity and flow rates.^284^ Flow not only affects margination of circulating cells, but can also lead to upregulation of endothelial receptors and cytokines in response to changes in shear stress. This is particularly true for ICAM‐1, VCAM‐1, and IL‐1β.^285^, ^286^ Endothelial integrins are also sensitive to changes in blood flow and become activated in response to increased shear stress.^287^, ^288^ Blood flow is thus an important parameter to consider in studies of IE adhesion. A variety of in vitro assays have yielded important insights in this regard.^289^, ^290^, ^291^, ^292^, ^293^, ^294^, ^295^, ^296^ As an example, CD36‐specific adhesion of normal and ovalocytotic IEs were similar in static assays, but were markedly different in assays conducted under physiologically more plausible flow conditions.^297^ With respect to CM‐relevant and PfEMP1‐specific adhesion, flow‐based studies of adhesion of IEs expressing PfEMP1 variants that can mediate binding to both ICAM‐1 and EPCR have revealed synergies that could not be discerned in static assays.^14^, ^298^

### 4.4 Converging on the protein C pathway

In spite of the very significant morbidity and high mortality of cerebral *P*.* falciparum* malaria, the pathophysiology of the disease is only partly understood.^104^, ^191^, ^299^ A range of potential and non‐exclusive pathogenic mechanisms has been proposed, such as circulatory obstruction by sequestered IEs, imbalanced cytokine responses, and endothelial dysfunction and loss of BBB integrity.^105^, ^107^, ^108^, ^300^ The available evidence is slowly converging on a scenario where CM is the consequence of the impact of IEs expressing particular PfEMP1 variants on the protein C‐dependent maintenance of the integrity of brain endothelium.^17^, ^301^

**Figure 4:**
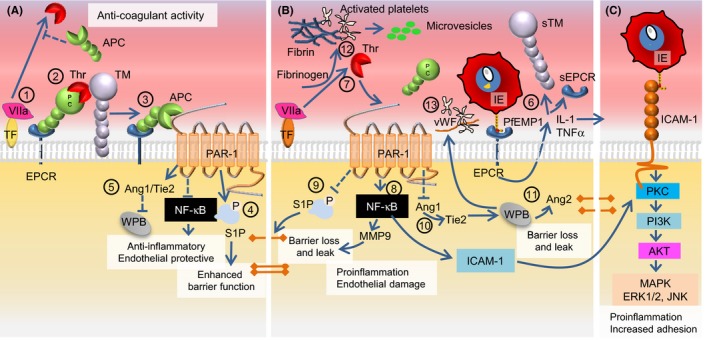
Linking the protein C pathway with EPCR‐ and ICAM‐1‐binding IEs in cerebral malaria. (A), Effects of EPCR in the absence of *Plasmodium falciparum*‐IE. Thrombin (Thr) is produced by the interaction between tissue factor (TF) and circulating activated factor VII (VIIa) (1). Thrombin initiates the EPCR‐ and thrombomodulin (TM)‐facilitated activation of protein C (APC) that then inhibits thrombin production (2). APC uses EPCR as a coreceptor for cleavage of proteinase‐activated receptor 1 (PAR‐1). The EPCR‐APC activation of PAR‐1 inhibits the nuclear factor‐κB pathway and exerts anti‐inflammatory and anti‐apoptotic activity (3). S1P signaling results in decreased endothelial permeability, and S1P production leads to enhancement of tight junctions and protection of endothelial barrier integrity (4). Angiopoetin‐1 (Ang1) produced in response to the APC‐PAR‐1 interaction decreases Weibel‐Palade body (WPB) exocytosis by occupying Tie2 (5). (B), The impact of infected erythrocytes expressing EPCR‐bound PfEMP1 on the surface. The IE‐EPCR interaction activates endothelial cells to release pro‐inflammatory cytokines (IL‐1, TNFα) that induce shedding of EPCR and TM from the endothelial surface and increases expression of ICAM‐1 (6). The EPCR‐IE interaction results in reduced levels of APC and increased thrombin generation with fibrin deposition (7). Increased levels of thrombin shift the PAR‐1 response toward activation of the RhoA and NFκB with increased surface expression of ICAM‐1 on the endothelial cell (8). The shift in the PAR‐1 response inhibits S1P release resulting in loss of tight junctions, and compromises endothelial barrier function by causing localized vascular leaks (9). A reduction in Ang‐1 levels increases WPB exocytosis via Tie2 and production of von Willebrand Factor (vWF) and Ang2 (10). Increased levels of Ang‐2 further increase WPB exocytosis and contribute to the loss of endothelial barrier integrity and leakage (11). Platelets become activated by thrombin and cytokines, which leads to production of platelet microvesicles (12). Thrombin and activated platelets combine to form thrombi (13). Strings of vWF and activated platelets form complexes, which like thrombi impair the cerebral circulation. (C), The increase in ICAM‐1 (in panel B) allows IE expressing PfEMP1 with a shared DBLβ ICAM‐1 motif to adhere to the brain endothelium. A large proportion of the ICAM‐1‐adhering IEs might initially bind EPCR via their CIDRα1 domains

The protein C pathway is a crucial anti‐coagulant and anti‐inflammatory regulator of thrombin production during clot formation.^302^ Normally (Figure 4), thrombomodulin on the endothelial surface binds thrombin and activates protein C to become activated protein C (APC) in a process that is strongly promoted by EPCR (also known as activated protein C receptor).^301^ The binding of APC to EPCR inhibits endothelial activation and TNFα‐dependent inflammation, thereby limiting the opportunity for IE sequestration mediated by known PfEMP1 receptors such as ICAM‐1, VCAM‐1, E‐selectin, and thrombospondin‐1.^303^ The interaction also activates PAR‐1, which has an anti‐apoptotic effect that protects the endothelial barrier integrity.^302^, ^304^

It has been proposed that this delicate system of checks‐and‐balances may be upset by IEs adhering to EPCR, thereby preventing physiologically appropriate activation of protein C.^301^, ^305^, ^306^ The PfEMP1 proteins expressed by EPCR‐adhering IEs bind EPCR near/at the site where protein C/APC normally binds. The IEs might thereby interfere with binding of the normal ligand and compromise protective APC‐dependent maintenance of the BBB via PAR‐1.^13^, ^307^, ^308^ The result would be excessive endothelial inflammation, thrombin activation, fibrin cross‐linking, platelet activation, upregulation of ICAM‐1 expression, downregulation of EPCR, and endothelial leakage. This corresponds to the petechial lesions, fibrin clots, EPCR denuding, thrombomodulin deficiency, axonal injury, and brain swelling that have been reported in pathology studies of the brains of patients who died of CM.^82^, ^301^, ^309^, ^310^, ^311^, ^312^, ^313^, ^314^, ^315^ The lack of APC may furthermore allow induction of endothelial dysfunction via parasite and host soluble factors, such as histones, heme, and HRP‐2, released locally when IEs rupture.^316^, ^317^

Brain microvascular endothelium may be particularly susceptible to the disruption of the protein C pathway by EPCR‐adhering IEs because EPCR is expressed at low levels at this site as opposed to the high expression seen in arteries and veins.^318^, ^319^ CM is associated with loss of EPCR in the brain, and increased levels of soluble EPCR have been reported and associated with CM mortality.^301^, ^320^ Human genetic variability affects the level of soluble EPCR, and there is some indication that some variants may be associated with protection from severe malaria, including CM, conceivably by neutralizing IE adhesion to EPCR.^232^ However, other studies did not detect such associations.^233^, ^234^ Substantial variation also exists in genetic sequence of the CIDR1α domains in PfEMP1 that mediate binding to EPCR, although the EPCR‐binding surface is largely conserved despite this variation.^13^ The variation nevertheless appears to impart differences in binding affinity that may affect how IE binding impacts normal EPCR function. The CIDRα1.1 domains in DC8 thus affected APC and thrombin‐induced permeability less than the CIDRα1.4 domains of DC13.^307^, ^321^ It is plausible that such diversity might contribute to the divergent pathophysiology of CM1 and CM2, where CM1 is characterized solely by IE sequestration, whereas CM2 also involves activation of coagulation and formation of fibrin clots and ring hemorrhages.^75^, ^82^, ^96^

The impact of EPCR‐binding IEs on the protein C pathway proposed above is consistent with features of CM pathogenesis, but most studies have found this IE adhesion phenotype to be associated with severe malaria in general, rather than with CM specifically.^209^, ^215^, ^216^, ^217^, ^226^, ^227^ However, this lack of specificity can be explained if CM pathogenesis requires parasites that express PfEMP1 variants that bind both to EPCR and ICAM‐1 (the abovementioned “double binders”). Our hypothesis involves a pathogenic cascade (Figure 4), where IEs initially adhere to EPCR on non‐inflamed cerebral endothelium.^14^, ^17^ This activates the endothelial cells as described above, induces their release of pro‐inflammatory cytokines, and increases their expression of ICAM‐1.^110^, ^219^, ^301^ “Double binders” may directly exploit this inflammatory response by adhering to ICAM‐1, and this has been associated with disruption of the BBB.^132^ In contrast, erythrocytes infected by parasites expressing PfEMP1 variants that only have affinity for EPCR are likely to dislodge, as EPCR is shed as a part of the inflammatory response.^301^

## 5 PfEMP1 AND IMMUNITY TO CM

In areas with stable transmission of *P*.* falciparum* parasites, susceptibility to clinical malaria is inversely correlated with age. Antibodies to parasite antigens on the surface of IEs are important, even decisive, determinants of this relationship, including the gradually decreasing risk of developing severe malaria such as CM.^322^, ^323^, ^324^, ^325^ Acquisition of this type of immunity following natural parasite exposure is remarkably slow, incomplete, and temporally unstable, characteristics that all point to variant antigens, and in particular to PfEMP1 as the primary antigenic target.^17^, ^326^

### 5.1 Naturally acquired, PfEMP1‐specific immunity

The variant‐specific, PfEMP1‐centric hypothesis of susceptibility to, and acquired immunological protection against, *P*.* falciparum* malaria hinges on the idea that the infecting parasites adapt to pre‐existing and developing immunity by switching to variants that are not recognized by specific antibodies.^327^, ^328^ Severe disease ensues when those variants enable IEs to adhere to receptors that are widespread, allow strong IE adhesion, are expressed in critical tissues, and/or have vital functions. This is more likely to occur in individuals with little or no acquired immunity, and to involve variants that are relatively conserved among different parasite clones. As immunity to initially virulent variants is acquired, the parasites are steadily forced to express variants that are less virulent (more diverse, less likely to mediate firm IE adhesion, less likely to bind to widespread receptors, and more likely to be expressed in tissues where the consequences of IE sequestration are less serious).

Variant‐specific immunity is indeed acquired in the orderly fashion predicted by the above theory. Antibodies to relatively conserved (“common”) parasite antigens and protection from severe malaria are acquired first, followed by antibodies to more variant antigens associated with uncomplicated disease, and eventually by antibodies recognizing very diverse (“rare”) antigens expressed on the surface of IEs obtained from carriers of asymptomatic/subclinical infections.^329^, ^330^, ^331^, ^332^ Transcription of *var* genes and acquisition of PfEMP1‐specific IgG follows this pattern, and shapes what PfEMP1 variants are compatible with parasite survival in a given semi‐immune host.^207^, ^238^, ^333^ Thus, *var* gene transcription in children with limited pre‐existing immunity and severe disease is skewed toward Group A,^202^, ^204^, ^207^ supporting the idea that these genes encode PfEMP1 proteins with adhesion specificities that are optimal for multiplication of the asexual parasites in non‐immune hosts, and likely include binding properties that predispose to severe malaria. As children acquire Group A anti‐PfEMP1 antibodies over successive infections, the proportion of *var* genes in Group B and C, encoding PfEMP1 variants associated with uncomplicated disease gradually increases.^238^, ^333^, ^334^, ^335^ The clearest example of this type of relationship so far is the susceptibility to placental *P*.* falciparum* infection, and the acquisition of immunity to that particular form of severe malaria, as both depend on a particular type of PfEMP1 called VAR2CSA.^187^, ^188^, ^336^ However, there is every reason to assume that it applies to all forms of *P*.* falciparum* malaria, including CM.^17^

IgG antibodies to the Group A, DC4‐containing PfEMP1 that can mediate IE adhesion to both ICAM‐1 and EPCR are acquired early in life in areas with stable transmission of *P*.* falciparum,* and this acquisition is associated with protection from severe malaria, including CM.^202^, ^337^, ^338^ The same applies to IgG specific for the CIDR domains that mediate IE adhesion to EPCR,^214^, ^339^, ^340^ although divergent evidence also exists.^341^ A very recent study indicates that IgG to ICAM‐1‐binding PfEMP1 variants from Group B, which do not share the ICAM‐1‐binding motif with the Group A PfEMP1s (including DC4 variants) and that do not contain an EPCR‐binding CIDR domain are acquired only when Group A (“dual binding”)‐specific immunity is already in place.^342^ This study thus further underpins the theory of a PfEMP1 hierarchy modulated by host immunity.

The degree of IgG cross‐reactivity that transcends clone‐specific variability in key antigens is of obvious importance. Encouragingly, naturally acquired antibodies capable of inhibiting the interaction between ICAM‐1 and many ICAM‐1‐binding DBLβ domain variants were recently observed.^338^ The prevalence of IgG specific for Group A ICAM‐1‐binding domains furthermore appears to be higher than those specific for EPCR‐binding CIDRα1 domains, suggesting that the former antibody specificity is more cross‐reactive than the latter.^341^

### 5.2 PfEMP1‐based vaccination and other PfEMP1‐specific interventions against CM

An ideal malaria vaccine would elicit immunity that prevents infection of humans and leads to transmission elimination and global eradication of *P*.* falciparum*. While this goal is elusive at the present time, vaccines that prevent severe malaria illness, particularly in children and pregnant women, could constitute an alternative or interim strategy.

Current efforts to develop VAR2CSA‐based vaccines to prevent placental malaria is the most advanced example of a PfEMP1‐based approach to malaria vaccination, as several such vaccines are currently in clinical trials.^343^, ^344^, ^345^, ^346^, ^347^ However, it is conceivable that development of vaccines to prevent severe malaria in children, including CM, might be possible using a similar strategy.^193^ An obvious goal would be vaccines eliciting a broadly reactive antibody response preventing, and ideally reversing, adhesion of IEs to ICAM‐1 and EPCR, which appear as key receptors in CM pathogenesis. Vaccines designed to specifically target “double binders” would seem particularly attractive. Blocking of EPCR‐specific adhesion of IE by vaccination with relevant CIDRα1 domains would lead to protection not only against CM, but also against severe childhood malaria in general. Unfortunately, EPCR‐binding CIDRα1 domains show substantial interclonal sequence variation and experimental antibodies binding distant variants are difficult to generate and are rarely inhibitory.^348^, ^349^ This reduces the likelihood of natural induction of cross‐inhibitory IgG responses targeting the critical parts of CIDRα1 domains. In contrast, the highly conserved ICAM‐1 binding site of these “dual binding” PfEMP1 variants encourage future efforts to raise broadly cross‐reactive IgG antibody responses against such molecules using their ICAM‐1 binding DBLβ domain as part of a strategy to prevent death due to CM.^14^, ^338^

However, many questions remain unanswered. Aside from the uncertainty regarding the feasibility of making a vaccine with the desired qualities, it is unclear whether inhibition/reversal of ICAM‐1/EPCR‐specific IE adhesion would be sufficient to prevent CM. It is similarly unclear to what extent acquired clinical protection from CM requires neutralizing (IE adhesion‐blocking) antibodies, as opposed to opsonizing antibodies leading to preferential phagocytic and/or cytotoxic removal of IEs expressing particularly pathogenic PfEMP1 variants.

While efforts to overcome the obstacles in the development of a PfEMP1‐specific vaccine to protect against CM continue, other interventions should also be pursued. Unfortunately, adjunctive therapies (eg, glucocorticoids, anti‐TNFαs, iron chelators, osmotic regulators, anti‐oxidants, and glycosaminoglycans), aimed at protecting against CM‐related brain damage and neuronal injury, have so far been unsuccessful.^72^, ^350^, ^351^ Furthermore, current anti‐malarial drugs fail to reverse adhesion of IEs.^352^ This notwithstanding, our improved understanding of molecular and cellular basis of CM pathogenesis might encourage novel adjunctive therapies aimed at dislodging sequestered parasite by interfering with PfEMP1‐mediated IE adhesion. Along this line, soluble EPCR has been shown to inhibit adhesion of DC8‐expressing IEs and endothelial cells in vitro.^28^ The finding of a soluble EPCR variant that binds PfEMP1 without affecting protein C binding to EPCR supports the in vivo feasibility of this approach,^13^ although the very low off‐rates of the PfEMP1‐EPCR interaction may not allow release of IEs already bound.^308^ Finally, a monoclonal antibody has been reported to inhibit and reverse adhesion of IEs to ICAM‐1, including antibodies that affect “dual binding” PfEMP1 variants.^353^ However, the associated cost of this intervention will probably prevent its use in clinical practice.^354^

## 6 CONCLUSIONS AND THE WAY FORWARD

Our understanding of the molecular basis of the interactions between *P*.* falciparum* parasites and the humans they infect is progressing at a rapid pace. This is not least so for the tissue‐, organ‐, and receptor‐specific adhesion of IEs that act as major contributors to the pathogenesis of *P*.* falciparum* malaria, including the development of severe complications such as CM.

The critical importance of PfEMP1 for parasite survival makes these antigens major immune targets, and acquisition of PfEMP1‐specific antibodies indeed appears to be a central component of naturally acquired protection. Emulating and accelerating these responses through vaccination would therefore seem obvious. However, the flipside is that the importance of PfEMP1 to the parasites applies a strong selective pressure on them to evolve mechanisms to evade protective, PfEMP1‐specific immunity. The most prominent example of this is clonal antigenic variation, which undoubtedly evolved to delay and frustrate the acquisition of PfEMP1‐specific protective antibodies, thereby enabling chronic infections.^17^, ^326^, ^355^, ^356^, ^357^, ^358^ It is an important issue to ascertain precisely how, and at what level(s), this undermining operates. While it seems clear that interclonal variation in functionally important antibody epitopes is involved, it is essentially unknown to what extent clonal antigenic PfEMP1 variation is affecting important T‐cell‐dependent helper functions. Clonal antigenic variation apart, it is becoming increasingly clear that *P*.* falciparum* parasites have evolved a range of other strategies to evade PfEMP1‐specific immunity. Examples are antigen topology and cloaking, interference with antigen presentation, and subversion of soluble host molecules.^20^, ^277^, ^359^, ^360^, ^361^, ^362^

In conclusion, much has been learnt from years of research on CM pathogenesis and immunity, although a lot remains to be known. Because this malaria complication is such an important part of the intolerable obstacle that *P*.* falciparum* malaria constitutes to health and to economic progress and equality, we are obliged to continue this research.^193^, ^363^

## CONFLICTS OF INTEREST

The authors declare no competing interests.

## References

[1] Alano P , Carter R . Sexual differentiation in malaria parasites. Annu Rev Microbiol. 1990;44:429‐449. 2252389 10.1146/annurev.mi.44.100190.002241

[2] Bannister LH , Hopkins JM , Dluzewski AR , et al. The *Plasmodium falciparum* apical membrane antigen‐1 (PfAMA‐1) is translocated within micronemes along subpellicular microtubules during merozoite development. J Cell Sci. 2003;116:3825‐3834. 12902400 10.1242/jcs.00665

[3] Miller LH , Ackerman HC , Su XZ , Wellems TE . Malaria biology and disease pathogenesis: insights for new treatments. Nat Med. 2013;19:156‐167. 23389616 10.1038/nm.3073 PMC4783790

[4] Zanghi G , Vembar SS , Baumgarten S , et al. A specific PfEMP1 is expressed in *Plasmodium falciparum* sporozoites and plays a role in hepatocyte infection. Cell Rep. 2018;22:2951‐2963. 29539423 10.1016/j.celrep.2018.02.075 PMC5863040

[5] Gardner MJ , Hall N , Fung E , et al. Genome sequence of the human malaria parasite *Plasmodium falciparum* . Nature. 2002;419:498‐511. 12368864 10.1038/nature01097 PMC3836256

[6] Quadt KA , Barfod L , Andersen D , et al. The density of knobs on *Plasmodium falciparum*‐infected erythrocytes depends on developmental age and varies among isolates. PLoS ONE. 2012;7:e45658. 23029166 10.1371/journal.pone.0045658 PMC3447797

[7] Kriek N , Tilley L , Horrocks P , et al. Characterization of the pathway for transport of the cytoadherence‐mediating protein, PfEMP1, to the host cell surface in malaria parasite‐infected erythrocytes. Mol Microbiol. 2003;50:1215‐1227. 14622410 10.1046/j.1365-2958.2003.03784.x

[8] Rask TS , Hansen DA , Theander TG , Pedersen AG , Lavstsen T *Plasmodium falciparum* erythrocyte membrane protein 1 diversity in seven genomes ‐ divide and conquer. PLoS Comput Biol. 2010;6:e1000933. 20862303 10.1371/journal.pcbi.1000933 PMC2940729

[9] Baruch DI , Ma XC , Singh HB , Bi XH , Pasloske BL , Howard RJ . Identification of a region of PfEMP1 that mediates adherence of *Plasmodium falciparum* infected erythrocytes to CD36: conserved function with variant sequence. Blood. 1997;90:3766‐3775. 9345064

[10] Peterson DS , Miller LH , Wellems TE . Isolation of multiple sequences from the *Plasmodium falciparum* genome that encode conserved domains homologous to those in erythrocyte‐binding proteins. Proc Natl Acad Sci USA. 1995;92:7100‐7104. 7624377 10.1073/pnas.92.15.7100 PMC41479

[11] Smith JD , Subramanian G , Gamain B , Baruch DI , Miller LH . Classification of adhesive domains in the *Plasmodium falciparum* erythrocyte membrane protein 1 family. Mol Biochem Parasitol. 2000;110:293‐310. 11071284 10.1016/s0166-6851(00)00279-6

[12] Higgins MK . The structure of a chondroitin sulfate‐binding domain important in placental malaria. J Biol Chem. 2008;283:21842‐21846. 18550531 10.1074/jbc.C800086200 PMC2494935

[13] Lau CK , Turner L , Jespersen JS , et al. Structural conservation despite huge sequence diversity allows EPCR binding by the malaria PfEMP1 family. Cell Host Microbe. 2015;17:118‐129. 25482433 10.1016/j.chom.2014.11.007 PMC4297295

[14] Lennartz F , Adams Y , Bengtsson A , et al. Structure‐guided identification of a family of dual receptor‐binding PfEMP1 that is associated with cerebral malaria. Cell Host Microbe. 2017;21:403‐414. 28279348 10.1016/j.chom.2017.02.009 PMC5374107

[15] Lavstsen T , Salanti A , Jensen A , Arnot DE , Theander TG . Sub‐grouping of *Plasmodium falciparum* 3D7 var genes based on sequence analysis of coding and non‐coding regions. Malar J. 2003;2:27. 14565852 10.1186/1475-2875-2-27 PMC222925

[16] Kraemer SM , Smith JD . Evidence for the importance of genetic structuring to the structural and functional specialization of the *Plasmodium falciparum var* gene family. Mol Microbiol. 2003;50:1527‐1538. 14651636 10.1046/j.1365-2958.2003.03814.x

[17] Hviid L , Jensen AT . PfEMP1 ‐ A parasite protein family of key importance in *Plasmodium falciparum* malaria immunity and pathogenesis. Adv Parasitol. 2015;88:51‐84. 25911365 10.1016/bs.apar.2015.02.004

[18] Bengtsson A , Joergensen L , Rask TS , et al. A novel domain cassette identifies *Plasmodium falciparum* PfEMP1 proteins binding ICAM‐1 and is a target of cross‐reactive, adhesion‐inhibitory antibodies. J Immunol. 2013;190:240‐249. 23209327 10.4049/jimmunol.1202578 PMC3539686

[19] Brown A , Turner L , Christoffersen S , et al. Molecular architecture of a complex between an adhesion protein from the malaria parasite and intracellular adhesion molecule 1. J Biol Chem. 2013;288:5992‐6003. 23297413 10.1074/jbc.M112.416347 PMC3581401

[20] Stevenson L , Huda P , Jeppesen A , et al. Investigating the function of F_c_‐specific binding of IgM to *Plasmodium falciparum* erythrocyte membrane protein 1 mediating erythrocyte rosetting. Cell Microbiol. 2015;17:819‐831. 25482886 10.1111/cmi.12403 PMC4737123

[21] Srivastava A , Gangnard S , Round A , et al. Full‐length extracellular region of the var2CSA variant of PfEMP1 is required for specific, high‐affinity binding to CSA. Proc Natl Acad Sci USA. 2010;107:4884‐4889. 20194779 10.1073/pnas.1000951107 PMC2841952

[22] Clausen TM , Christoffersen S , Dahlback M , et al. Structural and functional insight into how the *Plasmodium falciparum* VAR2CSA protein mediates binding to chondroitin sulfate A in placental malaria. J Biol Chem. 2012;287:23332‐23345. 22570492 10.1074/jbc.M112.348839 PMC3390611

[23] Moxon CA , Grau GE , Craig AG . Malaria: modification of the red blood cell and consequences in the human host. Br J Haematol. 2011;154(6):670‐679. 21623767 10.1111/j.1365-2141.2011.08755.x PMC3557659

[24] Hommel M , David PH , Oligino LD . Surface alterations of erythrocytes in *Plasmodium falciparum* malaria. Antigenic variation, antigenic diversity, and the role of the spleen. J Exp Med. 1983;157:1137‐1148. 6187885 10.1084/jem.157.4.1137 PMC2186973

[25] Cranston HA , Boylan CW , Carroll GL , et al. *Plasmodium falciparum* maturation abolishes physiologic red cell deformability. Science. 1984;223:400‐403. 6362007 10.1126/science.6362007

[26] Ockenhouse CF , Tandon NN , Magowan C , Jamieson GA , Chulay JD . Identification of a platelet membrane glycoprotein as a *falciparum* malaria sequestration receptor. Science. 1989;243:1469‐1471. 2467377 10.1126/science.2467377

[27] Berendt AR , Simmons DL , Tansey J , Newbold CI , Marsh K . Intercellular adhesion molecule‐1 is an endothelial cell adhesion receptor for *Plasmodium falciparum* . Nature. 1989;341:57‐59. 2475784 10.1038/341057a0

[28] Turner L , Lavstsen T , Berger SS , et al. Severe malaria is associated with parasite binding to endothelial protein C receptor. Nature. 2013;498:502‐505. 23739325 10.1038/nature12216 PMC3870021

[29] Rogerson SJ , Chaiyaroj SC , Ng K , Reeder JC , Brown GV . Chondroitin sulfate A is a cell surface receptor for *Plasmodium falciparum*‐infected erythrocytes. J Exp Med. 1995;182:15‐20. 7790815 10.1084/jem.182.1.15 PMC2192085

[30] Fried M , Duffy PE . Adherence of *Plasmodium falciparum* to chondroitin sulphate A in the human placenta. Science. 1996;272:1502‐1504. 8633247 10.1126/science.272.5267.1502

[31] Carlson J , Wahlgren M *Plasmodium falciparum* erythrocyte rosetting is mediated by promiscuous lectin‐like interactions. J Exp Med. 1992;176:1311‐1317. 1402677 10.1084/jem.176.5.1311 PMC2119436

[32] Pober JS . Cytokine‐mediated activation of vascular endothelium. Physiology and pathology. Am J Pathol. 1988;133:426‐433. 2462353 PMC1880803

[33] Grau GE , Taylor TE , Molyneux ME , et al. Tumor necrosis factor and disease severity in children with falciparum malaria. New Engl J Med. 1989;320:1586‐1591. 2657427 10.1056/NEJM198906153202404

[34] Howell DP , Levin EA , Springer AL , et al. Mapping a common interaction site used by *Plasmodium falciparum* Duffy binding‐like domains to bind diverse host receptors. Mol Microbiol. 2008;67:78‐87. 18047571 10.1111/j.1365-2958.2007.06019.x

[35] Janes JH , Wang CP , Levin‐Edens E , et al. Investigating the host binding signature on the *Plasmodium falciparum* PfEMP1 protein family. PLoS Pathog. 2011;7:e1002032. 21573138 10.1371/journal.ppat.1002032 PMC3088720

[36] Robinson BA , Welch TL , Smith JD . Widespread functional specialization of *Plasmodium falciparum* erythrocyte membrane protein 1 family members to bind CD36 analysed across a parasite genome. Mol Microbiol. 2003;47:1265‐1278. 12603733 10.1046/j.1365-2958.2003.03378.x

[37] Hsieh FL , Turner L , Bolla JR , Robinson CV , Lavstsen T , Higgins MK . The structural basis for CD36 binding by the malaria parasite. Nat Commun. 2016;7:12837. 27667267 10.1038/ncomms12837 PMC5052687

[38] Salanti A , Staalsoe T , Lavstsen T , et al. Selective upregulation of a single distinctly structured *var* gene in CSA‐adhering *Plasmodium falciparum* involved in pregnancy‐associated malaria. Mol Microbiol. 2003;49:179‐191. 12823820 10.1046/j.1365-2958.2003.03570.x

[39] Duffy MF , Maier AG , Byrne TJ , et al. VAR2CSA is the principal ligand for chondroitin sulfate A in two allogeneic isolates of *Plasmodium falciparum* . Mol Biochem Parasitol. 2006;148:117‐124. 16631964 10.1016/j.molbiopara.2006.03.006

[40] Chen Q , Heddini A , Barragan A , Fernandez V , Pearce SF , Wahlgren M . The semiconserved head structure of *Plasmodium falciparum* erythrocyte membrane protein 1 mediates binding to multiple independent host receptors. J Exp Med. 2000;192:1‐10. 10880521 10.1084/jem.192.1.1 PMC1887712

[41] Avril M , Bernabeu M , Benjamin M , Brazier AJ , Smith JD . Interaction between endothelial protein C receptor and intercellular adhesion molecule 1 to mediate binding of Plasmodium falciparum‐infected erythrocytes to endothelial cells. mBio. 2016;7:e00615‐00616. 27406562 10.1128/mBio.00615-16 PMC4958245

[42] Adams Y , Kuhnrae P , Higgins MK , Ghumra A , Rowe JA . Rosetting *Plasmodium falciparum*‐infected erythrocytes bind to human brain microvascular endothelial cells *in vitro*, demonstrating a dual adhesion phenotype mediated by distinct *P falciparum* erythrocyte membrane protein 1 domains. Infect Immun. 2014;82:949‐959. 24343658 10.1128/IAI.01233-13 PMC3958005

[43] Joergensen L , Bengtsson DC , Bengtsson A , et al. Surface co‐expression of two different PfEMP1 antigens on single *Plasmodium falciparum*‐infected erythrocytes facilitates binding to ICAM1 and PECAM1. PLoS Pathog. 2010;6:e1001083. 20824088 10.1371/journal.ppat.1001083 PMC2932717

[44] Scherf A , Hernandez‐Rivas R , Buffet P , et al. Antigenic variation in malaria: *in situ* switching, relaxed and mutually exclusive transcription of *var* genes during intra‐erythrocytic development in *Plasmodium falciparum* . EMBO J. 1998;17:5418‐5428. 9736619 10.1093/emboj/17.18.5418 PMC1170867

[45] Miller LH , Good MF , Milon G . Malaria pathogenesis. Science. 1994;264:1878‐1883. 8009217 10.1126/science.8009217

[46] Deitsch KW , Dzikowski R . Variant gene expression and antigenic variation by malaria parasites. Annu Rev Microbiol. 2017;71(1):625‐641. 28697665 10.1146/annurev-micro-090816-093841

[47] Horrocks P , Pinches R , Christodoulou Z , Kyes SA , Newbold CI . Variable *var* transition rates underlie antigenic variation in malaria. Proc Natl Acad Sci USA. 2004;101:11129‐11134. 15256597 10.1073/pnas.0402347101 PMC503751

[48] Frank M , Dzikowski R , Amulic B , Deitsch K . Variable switching rates of malaria virulence genes are associated with chromosomal position. Mol Microbiol. 2007;64:1486‐1498. 17555435 10.1111/j.1365-2958.2007.05736.x PMC3634120

[49] Recker M , Buckee CO , Serazin A , et al. Antigenic variation in *Plasmodium falciparum* malaria involves a highly structured switching pattern. PLoS Pathog. 2011;7:e1001306. 21408201 10.1371/journal.ppat.1001306 PMC3048365

[50] Noble R , Christodoulou Z , Kyes S , Pinches R , Newbold CI , Recker M . The antigenic switching network of *Plasmodium falciparum* and its implications for the immuno‐epidemiology of malaria. Elife. 2013;2:e01074. 24062941 10.7554/eLife.01074 PMC3778436

[51] Luse SA , Miller LH *Plasmodium falciparum* malaria. Ultrastructure of parasitized erythrocytes in cardiac vessels. Am J Trop Med Hyg. 1971;20:655‐660. 4999241

[52] Watermeyer JM , Hale VL , Hackett F , et al. A spiral scaffold underlies cytoadherent knobs in *Plasmodium falciparum*‐infected erythrocytes. Blood. 2016;127:343‐351. 26637786 10.1182/blood-2015-10-674002 PMC4797390

[53] Cutts EE , Laasch N , Reiter DM , et al. Structural analysis of *P falciparum* KAHRP and PfEMP1 complexes with host erythrocyte spectrin suggests a model for cytoadherent knob protrusions. PLoS Pathog. 2017;13:e1006552. 28806784 10.1371/journal.ppat.1006552 PMC5570508

[54] Looker O , Blanch AJ , Liu B , et al. The knob protein KAHRP assembles into a ring‐shaped structure that underpins virulence complex assembly. PLoS Pathog. 2019;15:e1007761. 31071194 10.1371/journal.ppat.1007761 PMC6529015

[55] De Koning‐Ward TF , Dixon MW , Tilley L , Gilson PR . Plasmodium species: master renovators of their host cells. Nat Rev Microbiol. 2016;14(8):494–507. 27374802 10.1038/nrmicro.2016.79

[56] Oberli A , Slater LM , Cutts E , et al. A *Plasmodium falciparum* PHIST protein binds the virulence factor PfEMP1 and comigrates to knobs on the host cell surface. FASEB J. 2014;28:4420‐4433. 24983468 10.1096/fj.14-256057 PMC4202109

[57] Proellocks NI , Herrmann S , Buckingham DW , et al. A lysine‐rich membrane‐associated PHISTb protein involved in alteration of the cytoadhesive properties of *Plasmodium falciparum*‐infected red blood cells. FASEB J. 2014;28:3103‐3113. 24706359 10.1096/fj.14-250399

[58] Sanchez CP , Karathanasis C , Sanchez R , et al. Single‐molecule imaging and quantification of the immune‐variant adhesin VAR2CSA on knobs of *Plasmodium falciparum*‐infected erythrocytes. Commun Biol. 2019;2:172. 10.1038/s42003-019-0429-z PMC6506540 31098405

[59] Horrocks P , Pinches RA , Chakravorty SJ , et al. PfEMP1 expression is reduced on the surface of knobless *Plasmodium falciparum* infected erythrocytes. J Cell Sci. 2005;118:2507‐2518. 15923663 10.1242/jcs.02381

[60] Subramaniam KS , Skinner J , Ivan E , et al. HIV malaria co‐infection is associated with atypical memory B cell expansion and a reduced antibody response to a broad array of *Plasmodium falciparum* antigens in Rwandan adults. PLoS ONE. 2015;10:e0124412. 25928218 10.1371/journal.pone.0124412 PMC4415913

[61] Abdi AI , Hodgson SH , Muthui MK , et al. *Plasmodium falciparum* malaria parasite *var* gene expression is modified by host antibodies: longitudinal evidence from controlled infections of Kenyan adults with varying natural exposure. BMC Infect Dis. 2017;17:585. 28835215 10.1186/s12879-017-2686-0 PMC5569527

[62] Crabb BS , Cooke BM , Reeder JC , et al. Targeted gene disruption shows that knobs enable malaria‐infected red cells to cytoadhere under physiological shear stress. Cell. 1997;89:287‐296. 9108483 10.1016/s0092-8674(00)80207-x

[63] Glenister FK , Coppel RL , Cowman AF , Mohandas N , Cooke BM . Contribution of parasite proteins to altered mechanical properties of malaria‐infected red blood cells. Blood. 2002;99:1060‐1063. 11807013 10.1182/blood.v99.3.1060

[64] Rug M , Prescott SW , Fernandez KM , Cooke BM , Cowman AF . The role of KAHRP domains in knob formation and cytoadherence of *P falciparum*‐infected human erythrocytes. Blood. 2006;108:370‐378. 16507777 10.1182/blood-2005-11-4624 PMC1895844

[65] Fairhurst RM , Baruch DI , Brittain NJ , et al. Abnormal display of PfEMP‐1 on erythrocytes carrying haemoglobin C may protect against malaria. Nature. 2005;435:1117‐1121. 15973412 10.1038/nature03631

[66] Cholera R , Brittain NJ , Gillrie MR , et al. Impaired cytoadherence of *Plasmodium falciparum*‐infected erythrocytes containing sickle hemoglobin. Proc Natl Acad Sci USA. 2008;105:991‐996. 18192399 10.1073/pnas.0711401105 PMC2242681

[67] Cyrklaff M , Sanchez CP , Kilian N , et al. Hemoglobins S and C interfere with actin remodeling in *Plasmodium falciparum*‐infected erythrocytes. Science. 2011;334:1283‐1286. 22075726 10.1126/science.1213775

[68] Krause MA , Diakite SA , Lopera‐Mesa TM , et al. a‐thalassemia impairs the cytoadherence of *Plasmodium falciparum*‐infected erythrocytes. PLoS ONE. 2012;7:e37214. 22623996 10.1371/journal.pone.0037214 PMC3356384

[69] Severe malaria. Trop Med Int Health. 2014;19(Suppl 1):7‐131. 25214480 10.1111/tmi.12313_2

[70] Marsh K , Forster D , Waruiru C , et al. Indicators of life‐threatening malaria in African children. New Engl J Med. 1995;332:1399‐1404. 7723795 10.1056/NEJM199505253322102

[71] Idro R , Jenkins NE , Newton CR . Pathogenesis, clinical features, and neurological outcome of cerebral malaria. Lancet Neurol. 2005;4:827‐840. 16297841 10.1016/S1474-4422(05)70247-7

[72] Mishra SK , Newton CR . Diagnosis and management of the neurological complications of *falciparum* malaria. Nat Rev Neurol. 2009;5:189‐198. 19347024 10.1038/nrneurol.2009.23 PMC2859240

[73] World Health Organization . World malaria report 2018. 2018.

[74] Newton CR , Pasvol G , Winstanley PA , Warrell DA . Cerebral malaria: what is unarousable coma? Lancet. 1990;335:472. 10.1016/0140-6736(90)90703-8 1968190

[75] Taylor TE , Fu W , Carr RA , et al. Differentiating the pathologies of cerebral malaria by *postmortem* parasite counts. Nat Med. 2004;10:143‐145. 14745442 10.1038/nm986

[76] Reyburn H , Mbatia R , Drakeley C , et al. Association of transmission intensity and age with clinical manifestations and case fatality of severe *Plasmodium falciparum* malaria. JAMA. 2005;293:1461‐1470. 15784869 10.1001/jama.293.12.1461

[77] Roca‐Feltrer A , Carneiro I , Smith L , Armstrong Schellenberg JR , Greenwood B , Schellenberg D . The age patterns of severe malaria syndromes in sub‐Saharan Africa across a range of transmission intensities and seasonality settings. Malar J. 2010;9:282. 20939931 10.1186/1475-2875-9-282 PMC2992028

[78] Postels DG , Birbeck GL , Valim C , Mannor KM , Taylor TE . Seasonal differences in retinopathy‐negative versus retinopathy‐positive cerebral malaria. Am J Trop Med Hyg. 2013;88:315‐318. 23166194 10.4269/ajtmh.2012.12-0415 PMC3583323

[79] Postels DG , Birbeck GL . Cerebral malaria. Handb Clin Neurol. 2013;114:91‐102. 23829902 10.1016/B978-0-444-53490-3.00006-6

[80] Trang TT , Phu NH , Vinh H , et al. Acute renal failure in patients with severe *falciparum* malaria. Clin Infect Dis. 1992;15:874‐880. 1445988 10.1093/clind/15.5.874

[81] Newton CR , Taylor TE , Whitten RO . Pathophysiology of fatal falciparum malaria in African children. Am J Trop Med Hyg. 1998;58:673‐683. 9598460 10.4269/ajtmh.1998.58.673

[82] Wassmer SC , Taylor TE , Rathod PK , et al. Investigating the pathogenesis of severe malaria: a multidisciplinary and cross‐geographical approach. Am J Trop Med Hyg. 2015;93:42‐56. 26259939 10.4269/ajtmh.14-0841 PMC4574273

[83] Hawkes M , Elphinstone RE , Conroy AL , Kain KC . Contrasting pediatric and adult cerebral malaria: the role of the endothelial barrier. Virulence. 2013;4:543‐555. 23924893 10.4161/viru.25949 PMC5359751

[84] Murphy SC , Breman JG . Gaps in the childhood malaria burden in Africa: cerebral malaria, neurological sequelae, anemia, respiratory distress, hypoglycemia, and complications of pregnancy. Am J Trop Med Hyg. 2001;64:57‐67. 11425178 10.4269/ajtmh.2001.64.57

[85] Idro R , Marsh K , John CC , Newton CR . Cerebral malaria: mechanisms of brain injury and strategies for improved neurocognitive outcome. Pediatr Res. 2010;68:267‐274. 20606600 10.1203/PDR.0b013e3181eee738 PMC3056312

[86] Beare NA , Taylor TE , Harding SP , Lewallen S , Molyneux ME . Malarial retinopathy: a newly established diagnostic sign in severe malaria. Am J Trop Med Hyg. 2006;75:790‐797. 17123967 PMC2367432

[87] Harding SP , Lewallen S , Beare NA , Smith A , Taylor TE , Molyneux ME . Classifying and grading retinal signs in severe malaria. Trop Doct. 2006;36(Suppl 1):1‐13. 10.1258/004947506776315781 16600082

[88] Seydel KB , Kampondeni SD , Valim C , et al. Brain swelling and death in children with cerebral malaria. N Engl J Med. 2015;372:1126‐1137. 25785970 10.1056/NEJMoa1400116 PMC4450675

[89] Lewallen S , Bronzan RN , Beare NA , Harding SP , Molyneux ME , Taylor TE . Using malarial retinopathy to improve the classification of children with cerebral malaria. Trans R Soc Trop Med Hyg. 2008;102:1089‐1094. 18760435 10.1016/j.trstmh.2008.06.014 PMC3804549

[90] MacCormick IJ , Beare NA , Taylor TE , et al. Cerebral malaria in children: using the retina to study the brain. Brain. 2014;137:2119‐2142. 24578549 10.1093/brain/awu001 PMC4107732

[91] Prato M , D'Alessandro S , Van den Steen PE , et al. Natural haemozoin modulates matrix metalloproteinases and induces morphological changes in human microvascular endothelium. Cell Microbiol. 2011;13:1275‐1285. 21707906 10.1111/j.1462-5822.2011.01620.x

[92] Maude RJ , Dondorp AM , Abu Sayeed A , Day NP , White NJ , Beare NA . The eye in cerebral malaria: what can it teach us? Trans R Soc Trop Med Hyg. 2009;103:661‐664. 19100590 10.1016/j.trstmh.2008.11.003 PMC2700878

[93] Villaverde C , Namazzi R , Shabani E , Opoka RO , John CC . Clinical comparison of retinopathy‐positive and retinopathy‐negative cerebral malaria. Am J Trop Med Hyg. 2017;96:1176‐1184. 28138045 10.4269/ajtmh.16-0315 PMC5417214

[94] Lewallen S , Bakker H , Taylor TE , Wills BA , Courtright P , Molyneux ME . Retinal findings predictive of outcome in cerebral malaria. Trans R Soc Trop Med Hyg. 1996;90:144‐146. 8761574 10.1016/s0035-9203(96)90116-9

[95] Beare NA , Southern C , Chalira C , Taylor TE , Molyneux ME , Harding SP . Prognostic significance and course of retinopathy in children with severe malaria. Arch Ophthalmol. 2004;122:1141‐1147. 15302654 10.1001/archopht.122.8.1141

[96] Dorovini‐Zis K , Schmidt K , Huynh H , et al. The neuropathology of fatal cerebral malaria in malawian children. Am J Pathol. 2011;178:2146‐2158. 21514429 10.1016/j.ajpath.2011.01.016 PMC3081150

[97] Barrera V , Hiscott PS , Craig AG , et al. Severity of retinopathy parallels the degree of parasite sequestration in eye and brain in Malawian children with fatal cerebral malaria. J Infect Dis. 2014;211:1977‐1986. 25351204 10.1093/infdis/jiu592 PMC4442623

[98] Newton C , Peshu N , Kendall B , et al. Brain swelling and ischaemia in Kenyans with cerebral malaria. Arch Dis Child. 1994;70:281‐287. 8185359 10.1136/adc.70.4.281 PMC1029778

[99] Newton C , Marsh K , Peshu N , Kirkham FJ . Perturbations of cerebral hemodynamics in Kenyans with cerebral malaria. Pediatr Neurol. 1996;15:41‐49. 8858700 10.1016/0887-8994(96)00115-4

[100] Persidsky Y , Ramirez SH , Haorah J , Kanmogne GD . Blood‐brain barrier: structural components and function under physiologic and pathologic conditions. J Neuroimmune Pharmacol. 2006;1:223‐236. 18040800 10.1007/s11481-006-9025-3

[101] Obermeier B , Daneman R , Ransohoff RM . Development, maintenance and disruption of the blood‐brain barrier. Nat Med. 2013;19:1584‐1596. 24309662 10.1038/nm.3407 PMC4080800

[102] Noumbissi ME , Galasso B , Stins MF . Brain vascular heterogeneity: implications for disease pathogenesis and design of in vitro blood‐brain barrier models. Fluids Barriers CNS. 2018;15:12. 29688865 10.1186/s12987-018-0097-2 PMC5911972

[103] Brown H , Rogerson S , Taylor T , et al. Blood‐brain barrier function in cerebral malaria in Malawian children. Am J Trop Med Hyg. 2001;64:207‐213. 11442219 10.4269/ajtmh.2001.64.207

[104] Medana IM , Turner GD . Human cerebral malaria and the blood‐brain barrier. Int J Parasitol. 2006;36:555‐568. 16616145 10.1016/j.ijpara.2006.02.004

[105] Storm J , Craig AG . Pathogenesis of cerebral malaria ‐ inflammation and cytoadherence. Front Cell Infect Microbiol. 2014;4:100. 25120958 10.3389/fcimb.2014.00100 PMC4114466

[106] Turner G , Morrison H , Jones M , et al. An immunohistochemical study of the pathology of fatal malaria: evidence for widespread endothelial activation and a potential role for intercellular adhesion molecule‐1 in cerebral sequestration. Am J Pathol. 1994;145:1057‐1069. 7526692 PMC1887431

[107] Brown H , Hien TT , Day N , et al. Evidence of blood‐brain barrier dysfunction in human cerebral malaria. Neuropathol Appl Neurobiol. 1999;25:331‐340. 10476050 10.1046/j.1365-2990.1999.00188.x

[108] Ponsford MJ , Medana IM , Prapansilp P , et al. Sequestration and microvascular congestion are associated with coma in human cerebral malaria. J Infect Dis. 2012;205:663‐671. 22207648 10.1093/infdis/jir812 PMC3266137

[109] Susomboon P , Maneerat Y , Dekumyoy P , et al. Down‐regulation of tight junction mRNAs in human endothelial cells co‐cultured with Plasmodium falciparum‐infected erythrocytes. Parasitol Int. 2006;55:107‐112. 16388977 10.1016/j.parint.2005.11.054

[110] Tripathi AK , Sullivan DJ , Stins MF *Plasmodium falciparum*‐infected erythrocytes decrease the integrity of human blood‐brain barrier endothelial cell monolayers. J Infect Dis. 2007;195:942‐950. 17330783 10.1086/512083

[111] Abbott NJ . Astrocyte‐endothelial interactions and blood‐brain barrier permeability. J Anat. 2002;200:629‐638. 12162730 10.1046/j.1469-7580.2002.00064.x PMC1570746

[112] Gavard J , Patel V , Gutkind JS . Angiopoietin‐1 prevents VEGF‐induced endothelial permeability by sequestering Src through mDia. Dev Cell. 2008;14:25‐36. 18194650 10.1016/j.devcel.2007.10.019

[113] Conroy AL , Lafferty EI , Lovegrove FE , et al. Whole blood angiopoietin‐1 and ‐2 levels discriminate cerebral and severe (non‐cerebral) malaria from uncomplicated malaria. Malar J. 2009;8:295. 20003529 10.1186/1475-2875-8-295 PMC2806378

[114] Conroy AL , Phiri H , Hawkes M , et al. Endothelium‐based biomarkers are associated with cerebral malaria in Malawian children: a retrospective case‐control study. PLoS ONE. 2010;5:e15291. 21209923 10.1371/journal.pone.0015291 PMC3012131

[115] Erdman LK , Dhabangi A , Musoke C , et al. Combinations of host biomarkers predict mortality among Ugandan children with severe malaria: a retrospective case‐control study. PLoS ONE. 2011;6:e17440. 21364762 10.1371/journal.pone.0017440 PMC3045453

[116] Finney CA , Hawkes CA , Kain DC , et al. S1P is associated with protection in human and experimental cerebral malaria. Mol Med. 2011;17(7‐8):717–725. 21556483 10.2119/molmed.2010.00214 PMC3146616

[117] D'Alessandro S , Basilico N , Prato M . Effects of *Plasmodium falciparum*‐infected erythrocytes on matrix metalloproteinase‐9 regulation in human microvascular endothelial cells. Asian Pac J Trop Med. 2013;6:195‐199. 23375032 10.1016/S1995-7645(13)60022-X

[118] Howard RJ , Uni S , Aikawa M , et al. Secretion of a malarial histidine‐rich protein (Pf HRP II) from *Plasmodium falciparum*‐infected erythrocytes. J Cell Biol. 1986;103:1269‐1277. 3533951 10.1083/jcb.103.4.1269 PMC2114335

[119] Pal P , Daniels BP , Oskman A , Diamond MS , Klein RS , Goldberg DE . Plasmodium falciparum histidine‐rich protein II compromises brain endothelial barriers and may promote cerebral malaria pathogenesis. MBio. 2016;7(3):pii: e00617‐16. 10.1128/mBio.00617-16 PMC4959673 27273825

[120] Aikawa M , Iseki M , Barnwell JW , Taylor D , Oo MM , Howard RJ . The pathology of human cerebral malaria. Am J Trop Med Hyg. 1990;43(suppl):30–37. 2202227 10.4269/ajtmh.1990.43.30

[121] Seydel KB , Fox LL , Glover SJ , et al. Plasma concentrations of parasite histidine‐rich protein 2 distinguish between retinopathy‐positive and retinopathy‐negative cerebral malaria in Malawian children. J Infect Dis. 2012;206:309‐318. 22634877 10.1093/infdis/jis371 PMC3490698

[122] Combes V , El‐Assaad F , Faille D , Jambou R , Hunt NH , Grau GE . Microvesiculation and cell interactions at the brain‐endothelial interface in cerebral malaria pathogenesis. Prog Neurogibol. 2010;91:140‐151. 10.1016/j.pneurobio.2010.01.007 20117166

[123] Hviid L , Theander TG , Elhassan IM , Jensen JB . Increased plasma levels of soluble ICAM‐1 and ELAM‐1 (E‐selectin) during acute *Plasmodium falciparum* malaria. Immunol Lett. 1993;36:51‐58. 7688346 10.1016/0165-2478(93)90068-d

[124] Elhassan IM , Hviid L , Satti G , et al. Evidence of endothelial inflammation, T cell activation, and T cell reallocation in uncomplicated *Plasmodium falciparum* malaria. Am J Trop Med Hyg. 1994;51:372‐379. 7524374 10.4269/ajtmh.1994.51.372

[125] Jakobsen PH , Morris‐Jones S , Rønn A , et al. Increased plasma concentrations of sICAM‐1, sVCAM‐1 and sELAM‐1 in patients with *Plasmodium falciparum* or *P vivax* malaria and association with disease severity. Immunology. 1994;83:665‐669. 7533138 PMC1415057

[126] Adukpo S , Kusi KA , Ofori MF , et al. High plasma levels of soluble intercellular adhesion molecule (ICAM)‐1 are associated with cerebral malaria. PLoS ONE. 2013;8:e84181. 24386348 10.1371/journal.pone.0084181 PMC3873986

[127] Moxon CA , Chisala NV , Wassmer SC , et al. Persistent endothelial activation and inflammation after *Plasmodium falciparum* infection in Malawian children. J Infect Dis. 2014;209:610‐615. 24048963 10.1093/infdis/jit419 PMC3903368

[128] Viebig NK , Wulbrand U , Forster R , Andrews KT , Lanzer M , Knolle PA . Direct activation of human endothelial cells by *Plasmodium falciparum*‐infected erythrocytes. Infect Immun. 2005;73:3271‐3277. 15908351 10.1128/IAI.73.6.3271-3277.2005 PMC1111820

[129] Chakravorty SJ , Carret C , Nash GB , Ivens A , Szestak T , Craig AG . Altered phenotype and gene transcription in endothelial cells, induced by *Plasmodium falciparum*‐infected red blood cells: pathogenic or protective? Int J Parasitol. 2007;37:975‐987. 17383656 10.1016/j.ijpara.2007.02.006 PMC1906861

[130] Francischetti IM , Seydel KB , Monteiro RQ , et al. *Plasmodium falciparum*‐infected erythrocytes induce tissue factor expression in endothelial cells and support the assembly of multimolecular coagulation complexes. J Thromb Haemost. 2007;5:155‐165. 17002660 10.1111/j.1538-7836.2006.02232.x PMC2892312

[131] Bridges DJ , Bunn J , van Mourik JA , et al. Rapid activation of endothelial cells enables *Plasmodium falciparum* adhesion to platelet‐decorated von Willebrand factor strings. Blood. 2010;115:1472‐1474. 19897581 10.1182/blood-2009-07-235150 PMC2840836

[132] Jambou R , Combes V , Jambou MJ , Weksler BB , Couraud PO , Grau GE *Plasmodium falciparum* adhesion on human brain microvascular endothelial cells involves transmigration‐like cup formation and induces opening of intercellular junctions. PLoS Pathog. 2010;6:e1001021. 20686652 10.1371/journal.ppat.1001021 PMC2912387

[133] Silamut K , Phu NH , Whitty C , et al. A quantitative analysis of the microvascular sequestration of malaria parasites in the human brain. Am J Pathol. 1999;155:395‐410. 10433933 10.1016/S0002-9440(10)65136-X PMC1866852

[134] Tripathi AK , Sha W , Shulaev V , Stins MF , Sullivan DJ Jr . *Plasmodium falciparum*‐infected erythrocytes induce NF‐kB regulated inflammatory pathways in human cerebral endothelium. Blood. 2009;114:4243‐4252. 19713460 10.1182/blood-2009-06-226415 PMC2925626

[135] Zougbede S , Miller F , Ravassard P , et al. Metabolic acidosis induced by *Plasmodium falciparum* intraerythrocytic stages alters blood‐brain barrier integrity. J Cereb Blood Flow Metab. 2011;31:514‐526. 20683453 10.1038/jcbfm.2010.121 PMC3049507

[136] Wassmer SC , Moxon CA , Taylor T , Grau GE , Molyneux ME , Craig AG . Vascular endothelial cells cultured from patients with cerebral or uncomplicated malaria exhibit differential reactivity to TNF. Cell Microbiol. 2011;13:198‐209. 21029292 10.1111/j.1462-5822.2010.01528.x PMC3041929

[137] Mandala WL , Msefula CL , Gondwe EN , Drayson MT , Molyneux ME , MacLennan CA . Cytokine profiles in Malawian children presenting with uncomplicated malaria, severe malarial anemia, and cerebral malaria. Clin Vaccine Immunol. 2017;24. 10.1128/CVI.00533-16 PMC5382826 28122790

[138] N'dilimabaka N , Taoufiq Z , Zougbede S, et al. *P. falciparum* isolate‐specific distinct patterns of induced apoptosis in pulmonary and brain endothelial cells. PLoS ONE. 2014;9:e90692. 24686750 10.1371/journal.pone.0090692 PMC3970966

[139] Pino P , Vouldoukis I , Kolb JP , et al. *Plasmodium falciparum*‐infected erythrocyte adhesion induces caspase activation and apoptosis in human endothelial cells. J Infect Dis. 2003;187:1283‐1290. 12696008 10.1086/373992

[140] Taoufiq Z , Gay F , Balvanyos J , et al. Rho kinase inhibition in severe malaria: thwarting parasite‐induced collateral damage to endothelia. J Infect Dis. 2008;197:1062‐1073. 18419473 10.1086/528988

[141] Wilson NO , Huang MB , Anderson W , et al. Soluble factors from *Plasmodium falciparum*‐infected erythrocytes induce apoptosis in human brain vascular endothelial and neuroglia cells. Mol Biochem Parasitol. 2008;162:172‐176. 18848585 10.1016/j.molbiopara.2008.09.003 PMC2671222

[142] Turner GD , Van Chuong L , Mai NT , et al. Systemic endothelial activation occurs in both mild and severe malaria ‐ Correlating dermal microvascular endothelial cell phenotype and soluble cell adhesion molecules with disease severity. Am J Pathol. 1998;152:1477‐1487. 9626052 PMC1858439

[143] Matsushita K , Morrell CN , Lowenstein CJ . Sphingosine 1‐phosphate activates Weibel‐Palade body exocytosis. Proc Natl Acad Sci USA. 2004;101:11483‐11487. 15273282 10.1073/pnas.0400185101 PMC509226

[144] Armah H , Dodoo AK , Wiredu EK , et al. High‐level cerebellar expression of cytokines and adhesion molecules in fatal, paediatric, cerebral malaria. Ann Trop Med Parasitol. 2005;99:629‐647. 16212798 10.1179/136485905X51508

[145] Pino P , Taoufiq Z , Nitcheu J , Vouldoukis I , Mazier D . Blood‐brain barrier breakdown during cerebral malaria: suicide or murder? Thromb Haemost. 2005;94:336‐340. 16113823 10.1160/TH05-05-0354

[146] Schindler SM , Little JP , Klegeris A . Microparticles: a new perspective in central nervous system disorders. Biomed Res Int. 2014;2014:756327. 24860829 10.1155/2014/756327 PMC4000927

[147] Lovegrove FE , Tangpukdee N , Opoka RO , et al. Serum angiopoietin‐1 and ‐2 levels discriminate cerebral malaria from uncomplicated malaria and predict clinical outcome in african children. PLoS ONE. 2009;4:e4912. 19300530 10.1371/journal.pone.0004912 PMC2657207

[148] Maisonpierre PC , Suri C , Jones PF , et al. Angiopoietin‐2, a natural antagonist for Tie2 that disrupts *in vivo* angiogenesis. Science. 1997;277:55‐60. 9204896 10.1126/science.277.5322.55

[149] Fiedler U , Reiss Y , Scharpfenecker M , et al. Angiopoietin‐2 sensitizes endothelial cells to TNF‐a and has a crucial role in the induction of inflammation. Nat Med. 2006;12:235‐239. 16462802 10.1038/nm1351

[150] Yuan HT , Khankin EV , Karumanchi SA , Parikh SM . Angiopoietin 2 is a partial agonist/antagonist of Tie2 signaling in the endothelium. Mol Cell Biol. 2009;29:2011‐2022. 19223473 10.1128/MCB.01472-08 PMC2663314

[151] Jones N , Master Z , Jones J , et al. Identification of Tek/Tie2 binding partners. Binding to a multifunctional docking site mediates cell survival and migration. J Biol Chem. 1999;274:30896‐30905. 10521483 10.1074/jbc.274.43.30896

[152] Kim I , Moon SO , Park SK , Chae SW , Koh GY . Angiopoietin‐1 reduces VEGF‐stimulated leukocyte adhesion to endothelial cells by reducing ICAM‐1, VCAM‐1, and E‐selectin expression. Circ Res. 2001;89:477‐479. 11557733 10.1161/hh1801.097034

[153] Jones N , Chen SH , Sturk C , et al. A unique autophosphorylation site on Tie2/Tek mediates Dok‐R phosphotyrosine binding domain binding and function. Mol Cell Biol. 2003;23:2658‐2668. 12665569 10.1128/MCB.23.8.2658-2668.2003 PMC152553

[154] Saharinen P , Eklund L , Miettinen J , et al. Angiopoietins assemble distinct Tie2 signalling complexes in endothelial cell‐cell and cell‐matrix contacts. Nat Cell Biol. 2008;10:527‐537. 18425119 10.1038/ncb1715

[155] Conroy AL , Glover SJ , Hawkes M , et al. Angiopoietin‐2 levels are associated with retinopathy and predict mortality in Malawian children with cerebral malaria: a retrospective case‐control study. Crit Care Med. 2012;40:952‐959. 22343839 10.1097/CCM.0b013e3182373157 PMC3284252

[156] Bergmark B , Bergmark R , Beaudrap PD , et al. Inhaled nitric oxide and cerebral malaria: basis of a strategy for buying time for pharmacotherapy. Pediatr Infect Dis J. 2012;31:e250‐e254. 22760538 10.1097/INF.0b013e318266c113

[157] Anstey NM , Weinberg JB , Hassanali MY , et al. Nitric oxide in Tanzanian children with malaria: inverse relationship between malaria severity and nitric oxide production/nitric oxide synthase type 2 expression. J Exp Med. 1996;184:557‐567. 8760809 10.1084/jem.184.2.557 PMC2192721

[158] Lopansri BK , Anstey NM , Weinberg JB , et al. Low plasma arginine concentrations in children with cerebral malaria and decreased nitric oxide production. Lancet. 2003;361:676‐678. 12606182 10.1016/S0140-6736(03)12564-0

[159] Yeo TW , Lampah DA , Gitawati R , et al. Angiopoietin‐2 is associated with decreased endothelial nitric oxide and poor clinical outcome in severe falciparum malaria. Proc Natl Acad Sci USA. 2008;105:17097‐17102. 18957536 10.1073/pnas.0805782105 PMC2575222

[160] De Caterina R , Libby P , Peng HB , et al. Nitric oxide decreases cytokine‐induced endothelial activation. Nitric oxide selectively reduces endothelial expression of adhesion molecules and proinflammatory cytokines. J Clin Invest. 1995;96:60‐68. 7542286 10.1172/JCI118074 PMC185173

[161] Matsushita K , Morrell CN , Cambien B , et al. Nitric oxide regulates exocytosis by S‐nitrosylation of N‐ethylmaleimide‐sensitive factor. Cell. 2003;115:139‐150. 14567912 10.1016/s0092-8674(03)00803-1 PMC2846406

[162] Fukuhara S , Sako K , Minami T , et al. Differential function of Tie2 at cell‐cell contacts and cell‐substratum contacts regulated by angiopoietin‐1. Nat Cell Biol. 2008;10:513‐526. 18425120 10.1038/ncb1714

[163] Serirom S , Raharjo WH , Chotivanich K , Loareesuwan S , Kubes P , Ho M . Anti‐adhesive effect of nitric oxide on *Plasmodium falciparum* cytoadherence under flow. Am J Pathol. 2003;162:1651‐1660. 12707049 10.1016/S0002-9440(10)64299-X PMC1851209

[164] Mwanga‐Amumpaire J , Carroll RW , Baudin E , et al. Inhaled nitric oxide as an adjunctive treatment for cerebral malaria in children: a phase II randomized open‐label clinical trial. Open Forum Infect Dis. 2015;2:ofv111. 26309894 10.1093/ofid/ofv111 PMC4542141

[165] Valentijn KM , Sadler JE , Valentijn JA , Voorberg J , Eikenboom J . Functional architecture of Weibel‐Palade bodies. Blood. 2011;117:5033‐5043. 21266719 10.1182/blood-2010-09-267492 PMC3109530

[166] O'Sullivan JM , Preston RJ , O'Regan N , O'Donnell JS . Emerging roles for haemostatic dysfunction in malaria pathogenesis. Blood. 2016;127:2281‐2288. 26851291 10.1182/blood-2015-11-636464

[167] Pain A , Ferguson D , Kai O , et al. Platelet‐mediated clumping of *Plasmodium falciparum*‐infected erythrocytes is a common adhesive phenotype and is associated with severe malaria. Proc Natl Acad Sci USA. 2001;98:1805‐1810. 11172032 10.1073/pnas.98.4.1805 PMC29338

[168] Biswas AK , Hafiz A , Banerjee B , Kim KS , Datta K , Chitnis CE . *Plasmodium falciparum* uses gC1qR/HABP1/p32 as a receptor to bind to vascular endothelium and for platelet‐mediated clumping. PLoS Pathog. 2007;3:e130. 10.1371/journal.ppat.0030130 PMC2323294 17907801

[169] Wassmer SC , Taylor T , Maclennan CA , et al. Platelet‐induced clumping of *Plasmodium falciparum*‐infected erythrocytes from Malawian patients with cerebral malaria‐possible modulation in vivo by thrombocytopenia. J Infect Dis. 2008;197:72‐78. 18171288 10.1086/523761 PMC2570538

[170] Pongponratn E , Riganti M , Harinasuta T , Bunnag D . Electron microscopy of the human brain in cerebral malaria. Southeast Asian J Trop Med Public Health. 1985;16:219‐227. 3906917

[171] Wassmer SC , Combes V , Candal FJ , Juhan‐Vague I , Grau GE . Platelets potentiate brain endothelial alterations induced by *Plasmodium falciparum* . Infect Immun. 2006;74:645‐653. 16369021 10.1128/IAI.74.1.645-653.2006 PMC1346683

[172] Wassmer SC , de Souza JB , Frere C , Candal FJ , Juhan‐Vague I , Grau GE . TGF‐b1 released from activated platelets can induce TNF‐stimulated human brain endothelium apoptosis: a new mechanism for microvascular lesion during cerebral malaria. J Immunol. 2006;176:1180‐1184. 16394007 10.4049/jimmunol.176.2.1180

[173] Grau GE , Mackenzie CD , Carr RA , et al. Platelet accumulation in brain microvessels in fatal pediatric cerebral malaria. J Infect Dis. 2003;187:461‐466. 12552430 10.1086/367960

[174] Larkin D , de Laat B , Jenkins PV , et al. Severe *Plasmodium falciparum* malaria is associated with circulating ultra‐large von Willebrand multimers and ADAMTS13 inhibition. PLoS Pathog. 2009;5:e1000349. 19300493 10.1371/journal.ppat.1000349 PMC2652105

[175] Curtis AM , Edelberg J , Jonas R , et al. Endothelial microparticles: sophisticated vesicles modulating vascular function. Vasc Med. 2013;18:204‐214. 23892447 10.1177/1358863X13499773 PMC4437568

[176] Combes V , Taylor TE , Juhan‐Vague I , et al. Circulating endothelial microparticles in Malawian children with severe falciparum malaria complicated with coma. J Am Med Assoc. 2004;291:2542‐2544. 10.1001/jama.291.21.2542-b 15173142

[177] Pankoui Mfonkeu JB , Gouado I , Fotso KH , et al. Elevated cell‐specific microparticles are a biological marker for cerebral dysfunctions in human severe malaria. PLoS ONE. 2010;5:e13415. 20976232 10.1371/journal.pone.0013415 PMC2954805

[178] Wheway J , Latham SL , Combes V , Grau GE . Endothelial microparticles interact with and support the proliferation of T cells. J Immunol. 2014;193:3378‐3387. 25187656 10.4049/jimmunol.1303431 PMC4170003

[179] Sampaio NG , Emery S , Garnham A , et al. Extracellular vesicles from early‐stage *P falciparum*‐infected red blood cells contain PfEMP1 and induce transcriptional changes in human monocytes. Cell Microbiol. 2018;20:e12822. 29349926 10.1111/cmi.12822

[180] Sampaio NG , Eriksson EM , Schofield L . *Plasmodium falciparum* PfEMP1 modulates monocyte/macrophage transcription factor activation and cytokine and chemokine responses. Infect Immun. 2018;86:e00447‐17 10.1128/IAI.00447-17 29038124 PMC5736827

[181] Langreth SG , Reese RT . Antigenicity of the infected‐erythrocyte and merozoite surfaces in *falciparum* malaria. J Exp Med. 1979;150:1241‐1254. 91658 10.1084/jem.150.5.1241 PMC2185704

[182] Howard RJ , Barnwell JW , Rock EP , et al. Two approximately 300 kilodalton *Plasmodium falciparum* proteins at the surface membrane of infected erythrocytes. Mol Biochem Parasitol. 1988;27:207‐224. 3278227 10.1016/0166-6851(88)90040-0

[183] Baruch DI , Pasloske BL , Singh HB , et al. Cloning the *P falciparum* gene encoding PfEMP1, a malarial variant antigen and adherence receptor on the surface of parasitized human erythrocytes. Cell. 1995;82:77‐87. 7541722 10.1016/0092-8674(95)90054-3

[184] Smith JD , Chitnis CE , Craig AG , et al. Switches in expression of *Plasmodium falciparum var* genes correlate with changes in antigenic and cytoadherent phenotypes of infected erythrocytes. Cell. 1995;82:101‐110. 7606775 10.1016/0092-8674(95)90056-x PMC3730239

[185] Su X , Heatwole VM , Wertheimer SP , et al. The large diverse gene family *var* encodes proteins involved in cytoadherence and antigenic variation of *Plasmodium falciparum*‐infected erythrocytes. Cell. 1995;82:89‐100. 7606788 10.1016/0092-8674(95)90055-1

[186] Bertin G , Ndam NT , Jafari‐Guemouri S , et al.Increased level of Plasmodium falciparum Pfcrt K76T mutation in pregnant women in Senegal. In: 2005. 10.1093/jac/dki097 15814601

[187] Fried M , Nosten F , Brockman A , Brabin BT , Duffy PE . Maternal antibodies block malaria. Nature. 1998;395:851‐852. 9804416 10.1038/27570

[188] Ricke CH , Staalsoe T , Koram K , et al. Plasma antibodies from malaria‐exposed pregnant women recognize variant surface antigens on *Plasmodium falciparum*‐infected erythrocytes in a parity‐dependent manner and block parasite adhesion to chondroitin sulphate A. J Immunol. 2000;165:3309‐3316. 10975848 10.4049/jimmunol.165.6.3309

[189] Salanti A , Dahlback M , Turner L , et al. Evidence for the involvement of VAR2CSA in pregnancy‐associated malaria. J Exp Med. 2004;200:1197‐1203. 15520249 10.1084/jem.20041579 PMC2211857

[190] Staalsoe T , Shulman CE , Bulmer JN , Kawuondo K , Marsh K , Hviid L . Variant surface antigen‐specific IgG and protection against the clinical consequences of pregnancy‐associated *Plasmodium falciparum* malaria. Lancet. 2004;363:283‐289. 14751701 10.1016/S0140-6736(03)15386-X

[191] Milner DA Jr , Whitten RO , Kamiza S , et al. The systemic pathology of cerebral malaria in African children. Front Cell Infect Microbiol. 2014;4:104. 25191643 10.3389/fcimb.2014.00104 PMC4139913

[192] Milner DA Jr , Lee JJ , Frantzreb C , et al. Quantitative assessment of multiorgan tissue sequestration in fatal pediatric cerebral malaria. J Infect Dis. 2015;212:1317‐1321. 25852120 10.1093/infdis/jiv205 PMC4577044

[193] Hviid L , Lavstsen T , Jensen AT . A vaccine targeted specifically to prevent cerebral malaria – is there hope? Expert Rev Vaccines. 2018;17(7):565–567. 29898617 10.1080/14760584.2018.1488591

[194] Miller LH . Distribution of mature trophozoites and schizonts of *Plasmodium falciparum* in the organs of *Aoutus trivirgatus*, the night monkey. Am J Trop Med Hyg. 1969;18:860‐865. 4982221 10.4269/ajtmh.1969.18.860

[195] MacPherson GG , Warrell MJ , White NJ , Looaresuwan S , Warrell DA . Human cerebral malaria. A quantitative ultrastructural analysis of parasitized erythrocyte sequestration. Am J Pathol. 1985;119:385‐401. 3893148 PMC1888001

[196] Bignami A , Bastianelli A . Observation of estivo‐autumnal malaria. Rif Med. 1889;63:1334‐1335.

[197] Rogerson SJ , Hviid L , Duffy PE , Leke R , Taylor DW . Malaria in pregnancy: pathogenesis and immunity. Lancet Infect Dis. 2007;7:105‐117. 17251081 10.1016/S1473-3099(07)70022-1

[198] Plewes K , Turner G , Dondorp AM . Pathophysiology, clinical presentation, and treatment of coma and acute kidney injury complicating *falciparum* malaria. Curr Opin Infect Dis. 2018;31:69‐77. 29206655 10.1097/QCO.0000000000000419 PMC5768231

[199] Brejt JA , Golightly LM . Severe malaria: update on pathophysiology and treatment. Curr Opin Infect Dis. 2019. 10.1097/QCO.0000000000000584 31369419

[200] Dondorp AM , Pongponratn E , White NJ . Reduced microcirculatory flow in severe falciparum malaria: pathophysiology and electron‐microscopic pathology. Acta Trop. 2004;89:309‐317. 14744557 10.1016/j.actatropica.2003.10.004

[201] Dondorp AM , Ince C , Charunwatthana P , et al. Direct *in vivo* assessment of microcirculatory dysfunction in severe *falciparum* malaria. J Infect Dis. 2008;197:79‐84. 18171289 10.1086/523762

[202] Jensen A , Magistrado PA , Sharp S , et al. *Plasmodium falciparum* associated with severe childhood malaria preferentially expresses PfEMP1 encoded by Group A *var* genes. J Exp Med. 2004;199:1179‐1190. 15123742 10.1084/jem.20040274 PMC2211911

[203] Bull PC , Berriman M , Kyes S , et al. *Plasmodium falciparum* variant surface antigen expression patterns during malaria. PLoS Pathog. 2005;1:e26. 16304608 10.1371/journal.ppat.0010026 PMC1287908

[204] Kyriacou HM , Stone GN , Challis RJ , et al. Differential *var* gene transcription in *Plasmodium falciparum* isolates from patients with cerebral malaria compared to hyperparasitaemia. Mol Biochem Parasitol. 2006;150:211‐218. 16996149 10.1016/j.molbiopara.2006.08.005 PMC2176080

[205] Rottmann M , Lavstsen T , Mugasa JP , et al. Differential expression of *var* gene groups is associated with morbidity caused by *Plasmodium falciparum* infection in Tanzanian children. Infect Immun. 2006;74:3904‐3911. 16790763 10.1128/IAI.02073-05 PMC1489729

[206] Falk N , Kaestli M , Qi W , et al. Analysis of *Plasmodium falciparum var* genes expressed in children from Papua New Guinea. J Infect Dis. 2009;200:347‐356. 19552523 10.1086/600071

[207] Warimwe G , Keane TM , Fegan G , et al. *Plasmodium falciparum var* gene expression is modified by host immunity. Proc Natl Acad Sci USA. 2009;106:21801‐21806. 20018734 10.1073/pnas.0907590106 PMC2792160

[208] Kalmbach Y , Rottmann M , Kombila M , Kremsner PG , Beck HP , Kun JF . Differential *var* gene expression in children with malaria and antidromic effects on host gene expression. J Infect Dis. 2010;202:313‐317. 20540611 10.1086/653586

[209] Lavstsen T , Turner L , Saguti F , et al. *Plasmodium falciparum* erythrocyte membrane protein 1 domain cassettes 8 and 13 are associated with severe malaria in children. Proc Natl Acad Sci USA. 2012;109:E1791‐E1800. 22619319 10.1073/pnas.1120455109 PMC3387094

[210] Warimwe GM , Fegan G , Musyoki JN , et al. Prognostic indicators of life‐threatening malaria are associated with distinct parasite variant antigen profiles. Sci Transl Med. 2012;4:129ra145. 10.1126/scitranslmed.3003247 PMC3491874 22496547

[211] Tembo DL , Nyoni B , Murikoli RV , et al. Differential PfEMP1 expression is associated with cerebral malaria pathology. PLoS Pathog. 2014;10:e1004537. 25473835 10.1371/journal.ppat.1004537 PMC4256257

[212] Bull PC , Buckee CO , Kyes S , et al. *Plasmodium falciparum* antigenic variation. Mapping mosaic *var* gene sequences onto a network of shared, highly polymorphic sequence blocks. Mol Microbiol. 2008;68:1519‐1534. 18433451 10.1111/j.1365-2958.2008.06248.x PMC2440560

[213] Avril M , Tripathi AK , Brazier AJ , et al. A restricted subset of *var* genes mediates adherence of *Plasmodium falciparum*‐infected erythrocytes to brain endothelial cells. Proc Natl Acad Sci USA. 2012;109:E1782‐E1790. 22619321 10.1073/pnas.1120534109 PMC3387091

[214] Claessens A , Adams Y , Ghumra A , et al. A subset of group A‐like *var* genes encodes the malaria parasite ligands for binding to human brain endothelial cells. Proc Natl Acad Sci USA. 2012;109:E1772‐E1781. 22619330 10.1073/pnas.1120461109 PMC3387129

[215] Jespersen JS , Wang CW , Mkumbaye SI , et al. *Plasmodium falciparum var* genes expressed in children with severe malaria encode CIDRa1 domains. EMBO Mol Med. 2016;8:839‐850. 27354391 10.15252/emmm.201606188 PMC4967939

[216] Bernabeu M , Danziger SA , Avril M , et al. Severe adult malaria is associated with specific PfEMP1 adhesion types and high parasite biomass. Proc Natl Acad Sci USA. 2016;113:E3270‐E3279. 27185931 10.1073/pnas.1524294113 PMC4988613

[217] Bertin GI , Lavstsen T , Guillonneau F , et al. Expression of the domain cassette 8 *Plasmodium falciparum* erythrocyte membrane protein 1 is associated with cerebral malaria in Benin. PLoS ONE. 2013;8:e68368. 23922654 10.1371/journal.pone.0068368 PMC3726661

[218] Cojean S , Jafari‐Guemouri S , Le Bras J , Durand R . Cytoadherence characteristics to endothelial receptors ICAM‐1 and CD36 of *Plasmodium falciparum* populations from severe and uncomplicated malaria cases. Parasite. 2008;15:163‐169. 18642510 10.1051/parasite/2008152163

[219] Tripathi AK , Sullivan DJ , Stins MF *Plasmodium falciparum*‐infected erythrocytes increase intercellular adhesion molecule 1 expression on brain endothelium through NF‐kB. Infect Immun. 2006;74:3262‐3270. 16714553 10.1128/IAI.01625-05 PMC1479273

[220] Newbold C , Warn P , Black G , et al. Receptor‐specific adhesion and clinical disease in *Plasmodium falciparum* . Am J Trop Med Hyg. 1997;57:389‐398. 9347951 10.4269/ajtmh.1997.57.389

[221] Rogerson SJ , Tembenu R , Dobaño C , Plitt S , Taylor TE , Molyneux ME . Cytoadherence characteristics of *Plasmodium falciparum*‐infected erythrocytes from Malawian children with severe and uncomplicated malaria. Am J Trop Med Hyg. 1999;61:467‐472. 10497992 10.4269/ajtmh.1999.61.467

[222] Ockenhouse CF , Ho M , Tandon NN , et al. Molecular basis of sequestration in severe and uncomplicated *Plasmodium falciparum* malaria: differential adhesion of infected erythrocytes to CD36 and ICAM‐1. J Infect Dis. 1991;164:163‐169. 1711552 10.1093/infdis/164.1.163

[223] Udomsangpetch R , Taylor BJ , Looareesuwan S , White NJ , Elliott JF , Ho M . Receptor specificity of clinical *Plasmodium falciparum* isolates: Nonadherence to cell‐bound E‐selectin and vascular cell adhesion molecule‐1. Blood. 1996;88:2754‐2760. 8839872

[224] Magistrado PA , Staalsoe T , Theander TG , Hviid L , Jensen AT . CD36 selection of 3D7 *Plasmodium falciparum* associated with severe childhood malaria results in reduced VAR4 expression. Malar J. 2008;7:204. 18844973 10.1186/1475-2875-7-204 PMC2572619

[225] Gray C , McCormick C , Turner G , Craig A . ICAM‐1 can play a major role in mediating *P falciparum* adhesion to endothelium under flow. Mol Biochem Parasitol. 2003;128:187‐193. 12742585 10.1016/s0166-6851(03)00075-6

[226] Mkumbaye SI , Wang CW , Lyimo E , et al. The severity of *Plasmodium falciparum* infection is associated with transcript levels of *var* genes encoding EPCR‐binding PfEMP1. Infect Immun. 2017;85:e00841‐16 28138022 10.1128/IAI.00841-16 PMC5364309

[227] Shabani E , Hanisch B , Opoka RO , Lavstsen T , John CC . Plasmodium falciparum EPCR‐binding PfEMP1 expression increases with malaria disease severity and is elevated in retinopathy negative cerebral malaria. BMC Med. 2017;15:183. 29025399 10.1186/s12916-017-0945-y PMC5639490

[228] Kessler A , Dankwa S , Bernabeu M , et al. Linking EPCR‐binding PfEMP1 to brain swelling in pediatric cerebral malaria. Cell Host Microbe. 2017;22(601–614):e605. 10.1016/j.chom.2017.09.009 PMC5783720 29107642

[229] Tuikue Ndam N , Moussiliou A , Lavstsen T , et al. Parasites causing cerebral falciparum malaria bind multiple endothelial receptors and express EPCR and ICAM‐1‐binding PfEMP1. J Infect Dis. 2017;215:1918‐1925. 28863469 10.1093/infdis/jix230

[230] Staalsoe T , Nielsen MA , Vestergaard LS , Jensen AT , Theander TG , Hviid L . *In vitro* selection of *Plasmodium falciparum* 3D7 for expression of variant surface antigens associated with severe malaria in African children. Parasite Immunol. 2003;25:421‐427. 14651589 10.1111/j.1365-3024.2003.00652.x

[231] Avril M , Brazier AJ , Melcher M , Sampath S , Smith JD . DC8 and DC13 *var* genes associated with severe malaria bind avidly to diverse endothelial cells. PLoS Pathog. 2013;9:e1003430. 23825944 10.1371/journal.ppat.1003430 PMC3694856

[232] Naka I , Patarapotikul J , Hananantachai H , Imai H , Ohashi J . Association of the endothelial protein C receptor (PROCR) rs867186‐G allele with protection from severe malaria. Malar J. 2014;13:105. 24635948 10.1186/1475-2875-13-105 PMC4004250

[233] Schuldt K , Ehmen C , Evans J , et al. Endothelial protein C receptor gene variants not associated with severe malaria in Ghanaian children. PLoS ONE. 2014;9:e115770. 25541704 10.1371/journal.pone.0115770 PMC4277309

[234] Hansson HH , Turner L , Moller L , et al. Haplotypes of the endothelial protein C receptor (EPCR) gene are not associated with severe malaria in Tanzania. Malar J. 2015;14:474. 26620701 10.1186/s12936-015-1007-6 PMC4666078

[235] Abdi AI , Kariuki SM , Muthui MK , et al. Differential *Plasmodium falciparum* surface antigen expression among children with malarial retinopathy. Sci Rep. 2015;5:18034. 26657042 10.1038/srep18034 PMC4677286

[236] Oleinikov AV , Amos E , Frye IT , et al. High throughput functional assays of the variant antigen PfEMP1 reveal a single domain in the 3D7 *Plasmodium falciparum* genome that binds ICAM1 with high affinity and is targeted by naturally acquired neutralizing antibodies. PLoS Pathog. 2009;5:e1000386. 19381252 10.1371/journal.ppat.1000386 PMC2663049

[237] Smith JD , Rowe JA , Higgins MK , Lavstsen T . Malaria's deadly grip: cytoadhesion of *Plasmodium falciparum*‐infected erythrocytes. Cell Microbiol. 2013;15:1976‐1983. 23957661 10.1111/cmi.12183 PMC3836831

[238] Cham CK , Turner L , Lusingu J , et al. Sequential, ordered acquisition of antibodies to *Plasmodium falciparum* erythrocyte membrane protein 1 domains. J Immunol. 2009;183:3356‐3363. 19675168 10.4049/jimmunol.0901331

[239] Ochola LB , Siddondo BR , Ocholla H , et al. Specific receptor usage in *Plasmodium falciparum* cytoadherence is associated with disease outcome. PLoS ONE. 2011;6:e14741. 21390226 10.1371/journal.pone.0014741 PMC3048392

[240] Cabrera A , Neculai D , Kain KC . CD36 and malaria: friends or foes? A decade of data provides some answers. Trends Parasitol. 2014;30:436‐444. 25113859 10.1016/j.pt.2014.07.006

[241] Esser C , Bachmann A , Kuhn D , et al. Evidence of promiscuous endothelial binding by *Plasmodium falciparum*‐infected erythrocytes. Cell Microbiol. 2014;16:701‐708. 24444337 10.1111/cmi.12270 PMC4114535

[242] David PH , Handunnetti SM , Leech JH , Gamage P , Mendis KN . Rosetting: a new cytoadherence property of malaria‐infected erythrocytes. Am J Trop Med Hyg. 1988;38:289‐297. 3354764 10.4269/ajtmh.1988.38.289

[243] Handunnetti SM , David PH , Perera K , Mendis KN . Uninfected erythrocytes form 'rosettes' around *Plasmodium falciparum* infected erythrocytes. Am J Trop Med Hyg. 1989;40:115‐118. 2645800 10.4269/ajtmh.1989.40.115

[244] Udomsangpetch R , Wåhlin B , Carlson J , et al. *Plasmodium falciparum*‐infected erythrocytes form spontaneous erythrocyte rosettes. J Exp Med. 1989;169:1835‐1840. 2654325 10.1084/jem.169.5.1835 PMC2189314

[245] Lowe BS , Mosobo M , Bull PC . All four species of human malaria parasites form rosettes. Trans R Soc Trop Med Hyg. 1998;92:526‐526. 9861369 10.1016/s0035-9203(98)90901-4

[246] Mercereau‐Puijalon O , Guillotte M , Vigan‐Womas I . Rosetting in *Plasmodium falciparum*: A cytoadherence phenotype with multiple actors. Transfus Clin Biol. 2008;15:62‐71. 18514562 10.1016/j.tracli.2008.04.003

[247] Rowe JA , Moulds JM , Newbold CI , Miller L *falciparum* rosetting mediated by a parasite‐variant erythrocyte membrane protein and complement‐receptor 1. Nature. 1997;388:292‐295. 9230440 10.1038/40888

[248] Barragan A , Kremsner PG , Wahlgren M , Carlson J . Blood group A antigen is a co‐receptor in *Plasmodium falciparum* rosetting. Infect Immun. 2000;68:2971‐2975. 10768996 10.1128/iai.68.5.2971-2975.2000 PMC97511

[249] Barragan A , Fernandez V , Chen Q , von Euler A , Wahlgren M , Spillmann D . The duffy‐binding‐like domain 1 of *Plasmodium falciparum* erythrocyte membrane protein 1 (PfEMP1) is a heparan sulfate ligand that requires 12 mers for binding. Blood. 2000;95:3594‐3599. 10828049

[250] Angeletti D , Sandalova T , Wahlgren M , Achour A . Binding of subdomains 1/2 of PfEMP1‐DBL1a to heparan sulfate or heparin mediates *Plasmodium falciparum* rosetting. PLoS ONE. 2015;10:e0118898. 25742651 10.1371/journal.pone.0118898 PMC4351205

[251] Chen Q , Barragan A , Fernandez V , et al. Identification of *Plasmodium falciparum* erythrocyte membrane protein 1 (PfEMP1) as the rosetting ligand of the malaria parasite *P falciparum* . J Exp Med. 1998;187:15‐23. 9419207 10.1084/jem.187.1.15 PMC2199182

[252] Goel S , Palmkvist M , Moll K , et al. RIFINs are adhesins implicated in severe *Plasmodium falciparum* malaria. Nat Med. 2015;21:314‐317. 25751816 10.1038/nm.3812

[253] Carlson J , Helmby H , Hill AV , Brewster D , Greenwood BM , Wahlgren M . Human cerebral malaria: association with erythrocyte rosetting and lack of anti‐rosetting antibodies. Lancet. 1990;336:1457‐1460. 1979090 10.1016/0140-6736(90)93174-n

[254] Treutiger C‐J , Hedlund I , Helmby H , et al. Rosette formation in *Plasmodium falciparum* isolates and anti‐rosette activity of sera from Gambians with cerebral or uncomplicated malaria. Am J Trop Med Hyg. 1992;46:503‐510. 1599043 10.4269/ajtmh.1992.46.503

[255] Wahlgren M , Carlson J , Helmby H , Hedlund I , Treutiger C‐J . Molecular mechanisms and biological importance of *Plasmodium falciparum* erythrocyte rosetting. Mem Inst Oswaldo Cruz. 1992;87:(suppl. III):323‐329. 10.1590/s0074-02761992000700054 1285315

[256] Carlson J . Erythrocyte rosetting in *Plasmodium falciparum* malaria ‐ with special reference to the pathogenesis of cerebral malaria. Scand J Infect Dis. 1993;86:1‐79. 8493454

[257] Vogt AM , Barragan A , Chen QJ , Kironde F , Spillmann D , Wahlgren M . Heparan sulfate on endothelial cells mediates the binding of *Plasmodium falciparum*‐infected erythrocytes via the DBL1a domain of PfEMP1. Blood. 2003;101:2405‐2411. 12433689 10.1182/blood-2002-07-2016

[258] Wang CW , Hviid L . Rifins, rosetting, and red blood cells. Trends Parasitol. 2015;31:285‐286. 25959958 10.1016/j.pt.2015.04.009

[259] Kaul DK , Roth EF Jr , Nagel RL , Howard RJ , Handunnetti SM . Rosetting of *Plasmodium falciparum*‐infected red blood cells with uninfected red blood cells enhances microvascular obstruction under flow conditions. Blood. 1991;78:812‐819. 1859893

[260] McCormick CJ , Craig A , Roberts D , Newbold CI , Berendt AR . Intercellular adhesion molecule‐1 and CD36 synergize to mediate adherence of *Plasmodium falciparum*‐infected erythrocytes to cultured human microvascular endothelial cells. J Clin Invest. 1997;100:2521‐2529. 9366566 10.1172/JCI119794 PMC508452

[261] Ringwald P , Peyron F , Lepers JP , et al. Parasite virulence factors during falciparum malaria: rosetting, cytoadherence, and modulation of cytoadherence by cytokines. Infect Immun. 1993;61:5198‐5204. 8225594 10.1128/iai.61.12.5198-5204.1993 PMC281301

[262] Rowe A , Obeiro J , Newbold CI , Marsh K *Plasmodium falciparum* rosetting is associated with malaria severity in Kenya. Infect Immun. 1995;63:2323‐2326. 7768616 10.1128/iai.63.6.2323-2326.1995 PMC173304

[263] Heddini A , Pettersson F , Kai O , et al. Fresh isolates from children with severe *Plasmodium falciparum* malaria bind to multiple receptors. Infect Immun. 2001;69:5849‐5856. 11500463 10.1128/IAI.69.9.5849-5856.2001 PMC98703

[264] Ho M , Davis T , Silamut K , Bunnag D , White NJ . Rosette formation of *Plasmodium falciparum*‐infected erythrocytes from patients with acute malaria. Infect Immun. 1991;59:2135‐2139. 2037374 10.1128/iai.59.6.2135-2139.1991 PMC257977

[265] Al‐Yaman F , Genton B , Mokela D , et al. Human cerebral malaria: lack of significant association between erythrocyte rosetting and disease severity. Trans R Soc Trop Med Hyg. 1995;89:55‐58. 7747308 10.1016/0035-9203(95)90658-4

[266] Angkasekwinai P , Looareesuwan S , Chaiyaroj SC . Lack of significant association between rosette formation and parasitized erythrocyte adherence to purified CD36. Southeast Asian J Trop Med Public Health. 1998;29:41‐45. 9740266

[267] Ho M , Singh B , Looaresuwan S , Davis T , Bunnag D , White NJ . Clinical correlates of *in vitro Plasmodium falciparum* cytoadherence. Infect Immun. 1991;59:873‐878. 1997437 10.1128/iai.59.3.873-878.1991 PMC258341

[268] Smith JD , Craig AG , Kriek N , et al. Identification of a *Plasmodium falciparum* intercellular adhesion molecule‐1 binding domain: a parasite adhesion trait implicated in cerebral malaria. Proc Natl Acad Sci USA. 2000;97:1766‐1771. 10677532 10.1073/pnas.040545897 PMC26510

[269] Rowe JA , Shafi J , Kai OK , Marsh K , Raza A . Nonimmune IgM, but not IgG binds to the surface of *Plasmodium falciparum*‐infected erythrocytes and correlates with rosetting and severe malaria. Am J Trop Med Hyg. 2002;66:692‐699. 12224576 10.4269/ajtmh.2002.66.692

[270] Clough B , Atilola FA , Black J , Pasvol G *Plasmodium falciparum*: the importance of IgM in the rosetting of parasite‐infected erythrocytes. Exp Parasitol. 1998;89:129‐132. 9603499 10.1006/expr.1998.4275

[271] Ghumra A , Semblat J‐P , McIntosh RS , et al. Identification of residues in the C4 domain of polymeric IgM essential for interaction with *Plasmodium falciparum* erythrocyte membrane protein 1 (PfEMP1). J Immunol. 2008;181:1988‐2000. 18641336 10.4049/jimmunol.181.3.1988 PMC2696179

[272] Scholander C , Treutiger CJ , Hultenby K , Wahlgren M . Novel fibrillar structure confers adhesive property to malaria‐ infected erythrocytes. Nat Med. 1996;2:204‐208. 8574966 10.1038/nm0296-204

[273] Somner EA , Black J , Pasvol G . Multiple human serum components act as bridging molecules in rosette formation by *Plasmodium falciparum*‐infected erythrocytes. Blood. 2000;95:674‐682. 10627479

[274] Akhouri RR , Goel S , Furusho H , Skoglund U , Wahlgren M . Architecture of human IgM in complex with *P falciparum* erythrocyte membrane protein 1. Cell Rep. 2016;14:723‐736. 26776517 10.1016/j.celrep.2015.12.067

[275] Jeppesen A , Ditlev SB , Soroka V , et al. Multiple *Plasmodium falciparum* erythrocyte membrane protein 1 variants per genome can bind IgM via its Fc fragment Fc. Infect Immun. 2015;83:3972‐3981. 26216422 10.1128/IAI.00337-15 PMC4567627

[276] Quintana M , Ecklu‐Mensah G , Tcherniuk SO , et al. Comprehensive analysis of Fc‐mediated IgM binding to the *Plasmodium falciparum* erythrocyte membrane protein 1 family in three parasite clones. Sci Rep. 2019;9:6050. 30988351 10.1038/s41598-019-42585-0 PMC6465264

[277] Stevenson L , Laursen E , Cowan GJ , et al. 2‐macroglobulin can crosslink multiple *Plasmodium falciparum* erythrocyte membrane protein 1 (PfEMP1) molecules and may facilitate adhesion of parasitized erythrocytes. PLoS Pathog. 2015;11:e1005022. 26134405 10.1371/journal.ppat.1005022 PMC4489720

[278] Mayor A , Hafiz A , Bassat Q , et al. Association of severe malaria outcomes with platelet‐mediated clumping and adhesion to a novel host receptor. PLoS ONE. 2011;6:e19422. 21559373 10.1371/journal.pone.0019422 PMC3084855

[279] Arman M , Raza A , Tempest LJ , et al. Platelet‐mediated clumping of *Plasmodium falciparum* infected erythrocytes is associated with high parasitemia but not severe clinical manifestations of malaria in African children. Am J Trop Med Hyg. 2007;77:943‐946. 17984358 PMC2696181

[280] Tangelder GJ , Slaaf DW , Muijtjens AM , Arts T . oude Egbrink MG , Reneman RS . Velocity profiles of blood platelets and red blood cells flowing in arterioles of the rabbit mesentery. Circ Res. 1986;59:505‐514. 3802426 10.1161/01.res.59.5.505

[281] Aarts PA , van den Broek SA , Prins GW , Kuiken GD , Sixma JJ , Heethaar RM . Blood platelets are concentrated near the wall and red blood cells, in the center in flowing blood. Arteriosclerosis. 1988;8:819‐824. 3196226 10.1161/01.atv.8.6.819

[282] Ahlqvist J . Decreased red cell deformability and vascular obstruction in *falciparum* malaria illustrated by a fatal case. Scand J Haematol. 1985;35:531‐535. 3911374 10.1111/j.1600-0609.1985.tb02824.x

[283] Dondorp AM , Nyanoti M , Kager PA , Mithwani S , Vreeken J , Marsh K . The role of reduced red cell deformability in the pathogenesis of severe falciparum malaria and its restoration by blood transfusion. Trans R Soc Trop Med Hyg. 2002;96:282‐286. 12174780 10.1016/s0035-9203(02)90100-8

[284] Chen Y , Li D , Li Y , Wan J , Li J , Chen H . Margination of stiffened red blood cells regulated by vessel geometry. Sci Rep. 2017;7:15253. 29127352 10.1038/s41598-017-15524-0 PMC5681636

[285] Tzima E , del Pozo MA , Shattil SJ , Chien S , Schwartz MA . Activation of integrins in endothelial cells by fluid shear stress mediates Rho‐dependent cytoskeletal alignment. EMBO J. 2001;20:4639‐4647. 11532928 10.1093/emboj/20.17.4639 PMC125600

[286] Davies PF . Hemodynamic shear stress and the endothelium in cardiovascular pathophysiology. Nat Clin Pract Cardiovasc Med. 2009;6:16‐26. 19029993 10.1038/ncpcardio1397 PMC2851404

[287] Wang Y , Miao H , Li S , et al. Interplay between integrins and FLK‐1 in shear stress‐induced signaling. Am J Physiol Cell Physiol. 2002;283:C1540‐C1547. 12372815 10.1152/ajpcell.00222.2002

[288] Chiu JJ , Chien S . Effects of disturbed flow on vascular endothelium: pathophysiological basis and clinical perspectives. Physiol Rev. 2011;91:327‐387. 21248169 10.1152/physrev.00047.2009 PMC3844671

[289] Cooke BM , Berendt AR , Craig AG , MacGregor J , Newbold CI , Nash GB . Rolling and stationary cytoadhesion of red blood cells parasitized by *Plasmodium falciparum*: separate roles for ICAM‐1, CD36 and thrombospondin. Br J Haematol. 1994;87:162‐170. 7524615 10.1111/j.1365-2141.1994.tb04887.x

[290] Cooke BM , Morris‐Jones S , Greenwood BM , Nash GB . Mechanisms of cytoadhesion of flowing, parasitized red blood cells from Gambian children with falciparum malaria. Am J Trop Med Hyg. 1995;53:29‐35. 7542844

[291] Cooke BM , Nash GB *Plasmodium falciparum*: Characterization of adhesion of flowing parasitized red blood cells to platelets. Exp Parasitol. 1995;80:116‐123. 7529716 10.1006/expr.1995.1013

[292] Udomsangpetch R , Reinhardt PH , Schollaardt T , Elliott JF , Kubes P , Ho M . Promiscuity of clinical *Plasmodium falciparum* isolates for multiple adhesion molecules under flow conditions. J Immunol. 1997;158:4358‐4364. 9126999

[293] Ho M , Schollaardt T , Niu X , Looareesuwan S , Patel KD , Kubes P . Characterization of *Plasmodium falciparum*‐infected erythrocyte and P‐selectin interaction under flow conditions. Blood. 1998;91:4803‐4809. 9616180

[294] Yipp BG , Anand S , Schollaardt T , Patel KD , Looareesuwan S , Ho M . Synergism of multiple adhesion molecules in mediating cytoadherence of *Plasmodium falciparum*‐infected erythrocytes to microvascular endothelial cells under flow. Blood. 2000;96:2292‐2298. 10979979

[295] Cooke BM , Glenister FK , Mohandas N , Coppel RL . Assignment of functional roles to parasite proteins in malaria‐infected red blood cells by competitive flow‐based adhesion assay. Br J Haematol. 2002;117:203‐211. 11918556 10.1046/j.1365-2141.2002.03404.x

[296] Cooke BM , Stuart J , Nash GB . The cellular and molecular rheology of malaria. Biorheology. 2014;51:99‐119. 24819866 10.3233/BIR-140654

[297] Cortes A , Mellombo M , Mgone CS , Beck HP , Reeder JC , Cooke BM . Adhesion of *Plasmodium falciparum*‐infected red blood cells to CD36 under flow is enhanced by the cerebral malaria‐protective trait South‐East Asian ovalocytosis. Mol Biochem Parasitol. 2005;142:252‐257. 15978955 10.1016/j.molbiopara.2005.03.016

[298] Bernabeu M , Gunnarsson C , Vishnyakova M , et al. Binding heterogeneity of Plasmodium falciparum to engineered 3D brain microvessels is mediated by EPCR and ICAM‐1. MBio. 2019;10(3):pii: e00420-19. 10.1128/mBio.00420-19 PMC6538777 31138740

[299] Van der Heyde HC , Nolan J , Combes V , Gramaglia I , Grau GE . A unified hypothesis for the genesis of cerebral malaria: sequestration, inflammation and hemostasis leading to microcirculatory dysfunction. Trends Parasitol. 2006;22:503‐508. 16979941 10.1016/j.pt.2006.09.002

[300] Grau GE , Craig AG . Cerebral malaria pathogenesis: revisiting parasite and host contributions. Future Microbiol. 2012;7:291‐302. 22324996 10.2217/fmb.11.155

[301] Moxon CA , Wassmer SC , Milner DA Jr , et al. Loss of endothelial protein C receptors links coagulation and inflammation to parasite sequestration in cerebral malaria in African children. Blood. 2013;122:842‐851. 23741007 10.1182/blood-2013-03-490219 PMC3731936

[302] Bouwens EA , Stavenuiter F , Mosnier LO . Mechanisms of anticoagulant and cytoprotective actions of the protein C pathway. J Thromb Haemost. 2013;11(Suppl 1):242‐253. 23809128 10.1111/jth.12247 PMC3713536

[303] Mosnier LO , Zlokovic BV , Griffin JH . The cytoprotective protein C pathway. Blood. 2007;109:3161‐3172. 17110453 10.1182/blood-2006-09-003004

[304] Stearns‐Kurosawa DJ , Kurosawa S , Mollica JS , Ferrell GL , Esmon CT . The endothelial cell protein C receptor augments protein C activation by the thrombin‐thrombomodulin complex. Proc Natl Acad Sci USA. 1996;93:10212‐10216. 8816778 10.1073/pnas.93.19.10212 PMC38363

[305] Aird WC , Mosnier LO , Fairhurst RM . Plasmodium falciparum picks (on) EPCR. Blood. 2014;123:163‐167. 24246501 10.1182/blood-2013-09-521005 PMC3888284

[306] Francischetti IM . Does activation of the blood coagulation cascade have a role in malaria pathogenesis? Trends Parasitol. 2008;24:258‐263. 18467176 10.1016/j.pt.2008.03.009 PMC2882796

[307] Gillrie MR , Avril M , Brazier AJ , et al. Diverse functional outcomes of *Plasmodium falciparum* ligation of EPCR: potential implications for malarial pathogenesis. Cell Microbiol. 2015;17:1883‐1899. 26119044 10.1111/cmi.12479 PMC4661070

[308] Petersen JE , Bouwens EA , Tamayo I , et al. Protein C system defects inflicted by the malaria parasite protein PfEMP1 can be overcome by a soluble EPCR variant. Thromb Haemost. 2015;114:1038‐1048. 26155776 10.1160/TH15-01-0018 PMC8142631

[309] Francischetti IM , Seydel KB , Monteiro RQ . Blood coagulation, inflammation, and malaria. Microcirculation. 2008;15:81‐107. 18260002 10.1080/10739680701451516 PMC2892216

[310] Dennis LH , Eichelberger JW , Inman MM , Conrad ME . Depletion of coagulation factors in drug‐resistant *Plasmodium falciparum* malaria. Blood. 1967;29:713‐721. 5337147

[311] Moxon CA , Heyderman RS , Wassmer SC . Dysregulation of coagulation in cerebral malaria. Mol Biochem Parasitol. 2009;166:99‐108. 19450727 10.1016/j.molbiopara.2009.03.006 PMC2724037

[312] Clemens R , Pramoolsinsap C , Lorenz R , Pukrittayakamee S , Bock HL , White NJ . Activation of the coagulation cascade in severe falciparum malaria through the intrinsic pathway. Br J Haematol. 1994;87:100‐105. 7947233 10.1111/j.1365-2141.1994.tb04877.x

[313] Mohanty D , Ghosh K , Nandwani SK , et al. Fibrinolysis, inhibitors of blood coagulation, and monocyte derived coagulant activity in acute malaria. Am J Hematol. 1997;54:23‐29. 8980257 10.1002/(sici)1096-8652(199701)54:1<23::aid-ajh4>3.0.co;2-6

[314] Holst FG , Hemmer CJ , Foth C , Seitz R , Egbring R , Dietrich M . Low levels of fibrin‐stabilizing factor (factor XIII) in human *Plasmodium falciparum* malaria: correlation with clinical severity. Am J Trop Med Hyg. 1999;60:99‐104. 9988331 10.4269/ajtmh.1999.60.99

[315] Medana IM , Day NP , Hien TT , et al. Axonal injury in cerebral malaria. Am J Pathol. 2002;160:655‐666. 11839586 10.1016/S0002-9440(10)64885-7 PMC1850649

[316] Gillrie MR , Lee K , Gowda DC , et al. *Plasmodium falciparum* histones induce endothelial proinflammatory response and barrier dysfunction. Am J Pathol. 2012;180:1028‐1039. 22260922 10.1016/j.ajpath.2011.11.037 PMC3448071

[317] Gallego‐Delgado J , Basu‐Roy U , Ty M , et al. Angiotensin receptors and b‐catenin regulate brain endothelial integrity in malaria. J Clin Invest. 2016;126:4016‐4029. 27643439 10.1172/JCI87306 PMC5096829

[318] Laszik Z , Mitro A , Taylor FB Jr , Ferrell G , Esmon CT . Human protein C receptor is present primarily on endothelium of large blood vessels: implications for the control of the protein C pathway. Circulation. 1997;96:3633‐3640. 9396465 10.1161/01.cir.96.10.3633

[319] Ye X , Fukudome K , Tsuneyoshi N , et al. The endothelial cell protein C receptor (EPCR) functions as a primary receptor for protein C activation on endothelial cells in arteries, veins, and capillaries. Biochem Biophys Res Commun. 1999;259:671‐677. 10364477 10.1006/bbrc.1999.0846

[320] Moussiliou A , Alao MJ , Denoeud‐Ndam L , et al. High plasma levels of soluble endothelial protein C receptor are associated with increased mortality among children with cerebral malaria in Benin. J Infect Dis. 2015;211:1484‐1488. 25425698 10.1093/infdis/jiu661

[321] Sampath S , Brazier AJ , Avril M , et al. *Plasmodium falciparum* adhesion domains linked to severe malaria differ in blockade of endothelial protein C receptor. Cell Microbiol. 2015;17:1868‐1882. 26118955 10.1111/cmi.12478 PMC4661071

[322] Lines J , Armstrong J . For a few parasites more: inoculum size, vector control and strain‐specific immunity to malaria. Parasitol Today. 1992;8:381‐383. 15463547 10.1016/0169-4758(92)90176-3

[323] Snow RW , Marsh K . The consequences of reducing transmission of *Plasmodium falciparum* in Africa. Adv Parasitol. 2002;52:235‐264. 12521262 10.1016/s0065-308x(02)52013-3

[324] Struik SS , Riley EM . Does malaria suffer from lack of memory? Immunol Rev. 2004;201:268‐290. 15361247 10.1111/j.0105-2896.2004.00181.x

[325] Smith TA , Leuenberger R , Lengeler C . Child mortality and malaria transmission intensity in Africa. Trends Parasitol. 2001;17:145‐149. 11286800 10.1016/s1471-4922(00)01814-6

[326] Hviid L . Naturally acquired immunity to *Plasmodium falciparum* malaria in Africa. Acta Trop. 2005;95:270‐275. 16018958 10.1016/j.actatropica.2005.06.012

[327] Marsh K , Howard RJ . Antigens induced on erythrocytes by *P falciparum*: expression of diverse and conserved determinants. Science. 1986;231:150‐153. 2417315 10.1126/science.2417315

[328] Bull PC , Lowe BS , Kortok M , Molyneux CS , Newbold CI , Marsh K . Parasite antigens on the infected red cell are targets for naturally acquired immunity to malaria. Nat Med. 1998;4:358‐360. 9500614 10.1038/nm0398-358 PMC3836255

[329] Bull PC , Lowe BS , Kortok M , Marsh K . Antibody recognition of *Plasmodium falciparum* erythrocyte surface antigens in Kenya: evidence for rare and prevalent variants. Infect Immun. 1999;67:733‐739. 9916084 10.1128/iai.67.2.733-739.1999 PMC96380

[330] Bull PC , Kortok M , Kai O , et al. *Plasmodium falciparum*‐infected erythrocytes: agglutination by diverse Kenyan plasma is associated with severe disease and young host age. J Infect Dis. 2000;182:252‐259. 10882604 10.1086/315652

[331] Nielsen MA , Staalsoe T , Kurtzhals J , et al. *Plasmodium falciparum* variant surface antigen expression varies between isolates causing severe and non‐severe malaria and is modified by acquired immunity. J Immunol. 2002;168:3444‐3450. 11907103 10.4049/jimmunol.168.7.3444

[332] Joergensen L , Vestergaard LS , Turner L , et al. 3D7‐derived *Plasmodium falciparum* erythrocyte membrane protein 1 is a frequent target of naturally acquired antibodies recognizing protein domains in a particular pattern independent of malaria transmission intensity. J Immunol. 2007;178:428‐435. 17182581 10.4049/jimmunol.178.1.428

[333] Cham GK , Turner L , Kurtis JD , et al. Hierarchical, domain type‐specific acquisition of antibodies to *Plasmodium falciparum* erythrocyte membrane protein 1 in Tanzanian children. Infect Immun. 2010;78:4653‐4659. 20823214 10.1128/IAI.00593-10 PMC2976311

[334] Ahmad KA , Wang G , Unger G , Slaton J , Ahmed K . Protein kinase CK2 ‐ a key suppressor of apoptosis. Adv Enzyme Regul. 2008;48:179‐187. 18492491 10.1016/j.advenzreg.2008.04.002 PMC2593134

[335] Duffy MF , Noviyanti R , Tsuboi T , et al. Differences in PfEMP1s recognized by antibodies from patients with uncomplicated or severe malaria. Malar J. 2016;15:258. 27149991 10.1186/s12936-016-1296-4 PMC4858840

[336] Hviid L , Salanti A . VAR2CSA and protective immunity against pregnancy‐associated *Plasmodium falciparum* malaria. Parasitology. 2007;134:1871‐1876. 17958922 10.1017/S0031182007000121

[337] Lusingu JP , Jensen AT , Vestergaard LS , et al. Levels of plasma immunoglobulin G with specificity against the cysteine‐rich interdomain regions of a semiconserved *Plasmodium falciparum* erythrocyte membrane protein 1, VAR4, predict protection against malarial anemia and febrile episodes. Infect Immun. 2006;74:2867‐2875. 16622225 10.1128/IAI.74.5.2867-2875.2006 PMC1459698

[338] Olsen RW , Ecklu‐Mensah G , Bengtsson A , et al. Natural and vaccine‐induced acquisition of cross‐reactive IgG‐inhibiting ICAM‐1‐specific binding of a *Plasmodium falciparum* PfEMP1 subtype associated specifically with cerebral malaria. Infect Immun. 2018;86:e00622‐17. 29426042 10.1128/IAI.00622-17 PMC5865037

[339] Turner L , Lavstsen T , Mmbando BP , et al. IgG antibodies to endothelial protein C receptor‐binding Cysteine‐rich interdomain region domains of *Plasmodium falciparum* erythrocyte membrane protein 1 are acquired early in life in individuals exposed to malaria. Infect Immun. 2015;83:3096‐3103. 26015475 10.1128/IAI.00271-15 PMC4496620

[340] Rambhatla JS , Turner L , Manning L , et al. Acquisition of antibodies against endothelial protein C receptor‐binding domains of *Plasmodium falciparum* erythrocyte membrane protein 1 in children with severe malaria. J Infect Dis. 2019;219:808‐818. 30365003 10.1093/infdis/jiy564

[341] Kessler A , Campo JJ , Harawa V , et al. Convalescent *Plasmodium falciparum*‐specific seroreactivity does not correlate with paediatric malaria severity or *Plasmodium* antigen exposure. Malar J. 2018;17:178. 29695240 10.1186/s12936-018-2323-4 PMC5918990

[342] Olsen RW , Ecklu‐Mensah G , Bengtsson A , et al. Acquisition of IgG to ICAM‐1‐binding DBL domains in the Plasmodium falciparum erythrocyte membrane protein 1 antigen family varies between Groups A, B and C. Infect Immun. 2019;pii:IAI.00224‐19 10.1128/IAI.00224-19. [Epub ahead of print]. PMC6759304 31308082

[343] Hviid L . Development of vaccines against *Plasmodium falciparum* malaria: taking lessons from naturally acquired protective immunity. Microbes Infect. 2007;9:772‐776. 17398137 10.1016/j.micinf.2007.02.008

[344] Hviid L . The case for PfEMP1‐based vaccines to protect pregnant women against *Plasmodium falciparum* malaria. Expert Rev Vaccines. 2011;10:1405‐1414. 21988306 10.1586/erv.11.113

[345] Pehrson C , Salanti A , Theander TG , Nielsen MA . Pre‐clinical and clinical development of the first placental malaria vaccine. Expert Rev Vaccines. 2017;16:613‐624. 28434376 10.1080/14760584.2017.1322512

[346] Mordmuller B , Sulyok M , Egger‐Adam D , et al. First‐in‐human, randomized, double‐blind clinical trial of differentially adjuvanted PAMVAC, a vaccine candidate to prevent pregnancy‐associated malaria. Clin Infect Dis. 2019. 10.1093/cid/ciy1140 PMC6792113 30629148

[347] Chene A , Gangnard S , Guadall A , et al. Preclinical immunogenicity and safety of the cGMP‐grade placental malaria vaccine PRIMVAC. EBioMedicine. 2019;42:145‐156. 30885725 10.1016/j.ebiom.2019.03.010 PMC6491931

[348] Turner L , Theander TG , Lavstsen T . Immunization with recombinant *P falciparum* erythrocyte membrane protein 1 CIDRa1 domains induces domain subtype inhibitory antibodies. Infect Immun. 2018;86:pii: e00435‐18. 10.1128/IAI.00435-18 PMC6204696 30150256

[349] Fougeroux C , Turner L , Bojesen AM , Lavstsen T , Holst PJ . Modified MHC class II‐associated invariant chain induces increased antibody responses against *Plasmodium falciparum* antigens after adenoviral vaccination. J Immunol. 2019;202:2320‐2331. 30833346 10.4049/jimmunol.1801210 PMC6452028

[350] Dondorp A , Nosten F , Stepniewska K , Day N , White N . Artesunate versus quinine for treatment of severe falciparum malaria: a randomised trial. Lancet. 2005;366:717‐725. 16125588 10.1016/S0140-6736(05)67176-0

[351] Hora R , Kapoor P , Thind KK , Mishra PC . Cerebral malaria ‐ clinical manifestations and pathogenesis. Metab Brain Dis. 2016;31:225‐237. 26746434 10.1007/s11011-015-9787-5

[352] Hughes KR , Biagini GA , Craig AG . Continued cytoadherence of *Plasmodium falciparum* infected red blood cells after antimalarial treatment. Mol Biochem Parasitol. 2010;169:71‐78. 19800372 10.1016/j.molbiopara.2009.09.007 PMC2814047

[353] Lennartz F , Bengtsson A , Olsen RW , et al. Mapping the binding site of a cross‐reactive *Plasmodium falciparum* PfEMP1 monoclonal antibody inhibitory of ICAM‐1 binding. J Immunol. 2015;195:3273‐3283. 26320251 10.4049/jimmunol.1501404 PMC4574524

[354] Goldring JP . Evaluation of immunotherapy to reverse sequestration in the treatment of severe *Plasmodium falciparum* malaria. Immunol Cell Biol. 2004;82:447‐452. 15283856 10.1111/j.0818-9641.2004.01265.x

[355] Collins WE , Jeffery GM . A retrospective examination of sporozoite‐ and trophozoite‐induced infections with *Plasmodium falciparum*: development of parasitologic and clinical immunity during primary infection. Am J Trop Med Hyg. 1999;61:4‐19. 10432041 10.4269/tropmed.1999.61-04

[356] Collins WE , Jeffery GM . A retrospective examination of secondary sporozoite‐ and trophozoite‐induced infections with *Plasmodium falciparum*: development of parasitologic and clinical immunity following secondary infection. Am J Trop Med Hyg. 1999;61:20‐35. 10432042 10.4269/tropmed.1999.61-020

[357] Collins WE , Jeffery GM . A retrospective examination of the patterns of recrudescence in patients infected with *Plasmodium falciparum* . Am J Trop Med Hyg. 1999;61:44‐48. 10432044 10.4269/tropmed.1999.61-044

[358] Staalsoe T , Hamad AA , Hviid L , Elhassan IM , Arnot DE , Theander TG *In vivo* switching between variant surface antigens in human *Plasmodium falciparum* infection. J Infect Dis. 2002;186:719‐722. 12195363 10.1086/342390

[359] Urban BC , Ferguson D , Pain A , et al. *Plasmodium falciparum*‐infected erythrocytes modulate the maturation of dendritic cells. Nature. 1999;400:73‐77. 10403251 10.1038/21900

[360] Barfod L , Dalgaard MB , Pleman ST , Ofori MF , Pleass RJ , Hviid L . Evasion of immunity to *Plasmodium falciparum* malaria by IgM masking of protective IgG epitopes in infected erythrocyte surface‐exposed PfEMP1. Proc Natl Acad Sci USA. 2011;108:12485‐12490. 21746929 10.1073/pnas.1103708108 PMC3145728

[361] Larsen MD , Quintana M , Ditlev SB , et al. Evasion of classical complement pathway activation on *Plasmodium falciparum*‐infected erythrocytes opsonized by PfEMP1‐specific IgG. Front Immunol. 2018;9:3088. 30666256 10.3389/fimmu.2018.03088 PMC6330326

[362] Rosa TF , Flammersfeld A , Ngwa CJ , et al. The *Plasmodium falciparum* blood stages acquire factor H family proteins to evade destruction by human complement. Cell Microbiol. 2016;18:573‐590. 26457721 10.1111/cmi.12535 PMC5063132

[363] Ashley EA , Pyae Phyo A , Woodrow CJ . Malaria. Lancet. 2018;391:1608‐1621. 29631781 10.1016/S0140-6736(18)30324-6

[364] White NJ , Turner GD , Medana IM , Dondorp AM , Day NP . The murine cerebral malaria phenomenon. Trends Parasitol. 2010;26:11‐15. 19932638 10.1016/j.pt.2009.10.007 PMC2807032

[365] Craig AG , Grau GE , Janse C , et al. The role of animal models for research on severe malaria. PLoS Pathog. 2012;8:e1002401. 22319438 10.1371/journal.ppat.1002401 PMC3271056

[366] Engwerda C , Belnoue E , Gruner AC , Renia L . Experimental models of cerebral malaria. Curr Top Microbiol Immunol. 2005;297:103‐143. 16265904

[367] Hearn J , Rayment N , Landon DN , Katz DR , De Souza JB . Immunopathology of cerebral malaria: morphological evidence of parasite sequestration in murine brain microvasculature. Infect Immun. 2000;68:5364‐5376. 10948166 10.1128/iai.68.9.5364-5376.2000 PMC101800

[368] Strangward P , Haley MJ , Shaw TN , et al. A quantitative brain map of experimental cerebral malaria pathology. PLoS Pathog. 2017;13:e1006267. 28273147 10.1371/journal.ppat.1006267 PMC5358898

[369] Grau GE , Tacchini‐Cottier F , Vesin C , et al. TNF‐induced microvascular pathology: active role for platelets and importance of the LFA‐1/ICAM‐1 interaction. Eur Cytokine Netw. 1993;4:415‐419. 7910490

[370] Hansen DS , Bernard NJ , Nie CQ , Schofield L . NK cells stimulate recruitment of CXCR3^+^ T cells to the brain during *Plasmodium berghei*‐mediated cerebral malaria. J Immunol. 2007;178:5779‐5788. 17442962 10.4049/jimmunol.178.9.5779

[371] Belnoue E , Potter SM , Rosa DS , et al. Control of pathogenic CD8^+^ T cell migration to the brain by IFN‐g during experimental cerebral malaria. Parasite Immunol. 2008;30:544‐553. 18665903 10.1111/j.1365-3024.2008.01053.x

[372] Nitcheu J , Bonduelle O , Combadiere C , et al. Perforin‐dependent brain‐infiltrating cytotoxic CD8^+^ T lymphocytes mediate experimental cerebral malaria pathogenesis. J Immunol. 2003;170:2221‐2228. 12574396 10.4049/jimmunol.170.4.2221

[373] Pongponratn E , Turner G , Day N , et al. An ultrastructural study of the brain in fatal *Plasmodium falciparum* malaria. Am J Trop Med Hyg. 2003;69:345‐359. 14640492

[374] Bauer PR , Van Der Heyde HC , Sun G , Specian RD , Granger DN . Regulation of endothelial cell adhesion molecule expression in an experimental model of cerebral malaria. Microcirculation. 2002;9:463‐470. 12483543 10.1038/sj.mn.7800159

[375] Engwerda CR , Mynott TL , Sawhney S , De Souza JB , Bickle QD , Kaye PM . Locally up‐regulated lymphotoxin a, not systemic tumor necrosis factor a, is the principle mediator of murine cerebral malaria. J Exp Med. 2002;195:1371‐1377. 12021316 10.1084/jem.20020128 PMC2193758

[376] Favre N , Da Laperousaz C , Ryffel B , et al. Role of ICAM‐1 (CD54) in the development of murine cerebral malaria. Microb Infect. 1999;1:961‐968. 10.1016/s1286-4579(99)80513-9 10617927

[377] Kwiatkowski D , Hill AV , Sambou I , et al. TNF concentration in fatal cerebral, non‐fatal cerebral, and uncomplicated *Plasmodium falciparum* malaria. Lancet. 1990;336:1201‐1204. 1978068 10.1016/0140-6736(90)92827-5

[378] Akanmori BD , Kurtzhals JA , Goka BQ , et al. Distinct patterns of cytokine regulation in discrete clinical forms of *Plasmodium falciparum* malaria. Eur Cytokine Netw. 2000;11:113‐118. 10705308

[379] Ghazanfari N , Mueller SN , Heath WR . Cerebral malaria in mouse and man. Front Immunol. 2018;9:2016. 30250468 10.3389/fimmu.2018.02016 PMC6139318

[380] Sierro F , Grau G . The ins and outs of cerebral malaria pathogenesis: Immunopathology, extracellular vesicles, immunometabolism, and trained immunity. Front Immunol. 2019;10:830. 31057552 10.3389/fimmu.2019.00830 PMC6478768

